# Disability Weights for Chronic Mercury Intoxication Resulting from Gold Mining Activities: Results from an Online Pairwise Comparisons Survey

**DOI:** 10.3390/ijerph14010057

**Published:** 2017-01-10

**Authors:** Nadine Steckling, Brecht Devleesschauwer, Julia Winkelnkemper, Florian Fischer, Bret Ericson, Alexander Krämer, Claudia Hornberg, Richard Fuller, Dietrich Plass, Stephan Bose-O’Reilly

**Affiliations:** 1 Unit Paediatric Environmental Epidemiology, WHO Collaborating Centre for Occupational Health, Institute and Outpatient Clinic for Occupational, Social and Environmental Medicine, University Hospital Munich, Ziemssenstr. 1, Munich 80336, Germany; stephan.boeseoreilly@med.uni-muenchen.de; 2 Department of Public Health, Health Services Research and Health Technology Assessment, Institute of Public Health, Medical Decision Making and Health Technology Assessment, UMIT—University for Health Sciences, Medical Informatics and Technology, Eduard Wallnoefer Center I, Hall in Tyrol 6060, Austria; 3 Department of Environment & Health, School of Public Health, Bielefeld University, Universitätsstr. 25, Bielefeld 33615, Germany; claudia.hornberg@uni-bielefeld.de; 4 Department of Public Health and Surveillance, Scientific Institute of Public Health (WIV-ISP), Rue Juliette Wytsmanstraat 14, Brussels 1050, Belgium; brechtdv@gmail.com; 5 Department of Public Health Medicine, School of Public Health, Bielefeld University, Universitätsstr. 25, Bielefeld 33615, Germany; julia.winkelnkemper@uni-bielefeld.de (J.W.); f.fischer@uni-bielefeld.de (F.F.); alexander.kraemer@uni-bielefeld.de (A.K.); 6 Pure Earth, Formerly Blacksmith Institute, 475 Riverside Drive, Suite 860, New York, NY 10115, USA; bret@pureearth.org (B.E.); fuller@pureearth.org (R.F.); 7 Section Exposure Assessment and Environmental Health Indicators, German Environment Agency, Corrensplatz 1, Berlin 14195, Germany; dietrich.plass@uba.de

**Keywords:** mercury, artisanal small-scale gold mining, chronic metallic mercury vapor intoxication, burden of disease, disability weights, pairwise comparison, visual analogue scale

## Abstract

In artisanal small-scale gold mining, mercury is used for gold-extraction, putting miners and nearby residents at risk of chronic metallic mercury vapor intoxication (CMMVI). Burden of disease (BoD) analyses allow the estimation of the public health relevance of CMMVI, but until now there have been no specific CMMVI disability weights (DWs). The objective is to derive DWs for moderate and severe CMMVI. Disease-specific and generic health state descriptions of 18 diseases were used in a pairwise comparison survey. Mercury and BoD experts were invited to participate in an online survey. Data were analyzed using probit regression. Local regression was used to make the DWs comparable to the Global Burden of Disease (GBD) study. Alternative survey (visual analogue scale) and data analyses approaches (linear interpolation) were evaluated in scenario analyses. A total of 105 participants completed the questionnaire. DWs for moderate and severe CMMVI were 0.368 (0.261–0.484) and 0.588 (0.193–0.907), respectively. Scenario analyses resulted in higher mean values. The results are limited by the sample size, group of interviewees, questionnaire extent, and lack of generally accepted health state descriptions. DWs were derived to improve the data basis of mercury-related BoD estimates, providing useful information for policy-making. Integration of the results into the GBD DWs enhances comparability.

## 1. Introduction

Artisanal small-scale gold mining (ASGM) is a poverty-driven activity practiced by an estimated 16 million gold miners worldwide [1]. Large quantities of mercury are used to extract gold from ore resulting in a high mercury exposure of miners and residents in ASGM areas [2]. Due to the informal and often illegal character [3], the public health burden of the mercury exposed gold miners has not been well studied. Steckling et al. [4] provided a preliminary estimate of the burden of disease (BoD) attributable to mercury exposure of gold miners in Zimbabwe resulting in an estimate of 95,400 Disability-Adjusted Life Years (DALYs). Pure Earth and Green Cross Switzerland [5] made an initial estimate of the global BoD due to ASGM, assuming 1.5 million attributable DALYs. One of the major limiting factors of these analyses was the lack of a disability weight (DW) for mercury intoxication, in addition to a generally scarce data basis. BoD analyses are useful to describe population health by integrating information about mortality and morbidity into a single metric, the Disability-Adjusted Life Year (DALY). Environmental burden of disease (EBD) is an application of BoD where the quantified burden is attributed to environmental risk factors. BoD and EBD generate information relevant for policy-making and prioritization. Indeed, DALYs allow comparing the impact of diseases and environmental factors across populations and across time [6]. The DALY is a summary measure of population health (SMPH) comprising the Years of Life Lost (YLL) and the Years Lived with Disability (YLD). YLLs are quantified by multiplying the number of deaths with a standard remaining life expectancy at age of death. YLDs are obtained by multiplying the number of incident cases with the average duration of disability and multiplied with the corresponding disability weight (DW) [7,8,9]. Alternatively, the number of prevalent cases can also be used as a measure of disease frequency, yielding a prevalence perspective for assessing BoD [10,11]. In any case, a DW is a crucial prerequisite for quantifying YLDs. The DW is a factor between zero and one reflecting the severity of the health state, defined as the relative reduction in quality of life. A value of zero designates an optimal health state (i.e., 0% reduction in quality of life), while a value of one indicates a health state equivalent to death (i.e., 100% reduction in quality of life). Consequently, a severe disease has a higher DW than a mild disease. Different approaches may be used to derive DWs [12]. DWs of more than 200 health states were recently derived within the Global Burden of Disease (GBD) study [13,14,15]. However, the list of health states without determined DW is still extensive. The absence of health state-specific DWs hinders a comparable estimation of the corresponding BoD. Consequently, the consideration in resource allocation is inhibited. This is the case for chronic intoxication due to the use of mercury in ASGM. The DiWIntox (Disability Weight for Chronic Mercury Intoxication) project was initiated to provide additional data in this research field. In the first stage of the project, disease profiles of moderate and severe chronic metallic mercury vapor intoxication (CMMVI) with generic and disease-specific descriptions useful for a DW derivation were determined by interviewing experts with extensive experiences in the field of mercury research and by conducting a systematic literature search [16]. A mild CMMVI was also confirmed by the experts. However, a differentiation in the two stages moderate and severe had advantages, while available information describes either CMMVI in general (what was labeled as the moderate case) or the most severe case of CMMVI [16]. The second stage of the project, described in this paper, aims to derive DWs for moderate and severe CMMVI following mercury exposures in ASGM. 

## 2. Materials and Methods

There is no standardized procedure for deriving DWs. However, numerous methodological approaches are available [12,17]. In general, DWs are based on valuation methods to raise the respondents preferences or the “relative desirability of a health state compared to” another ([12], p. 12, based on [18]). Important methodological decisions concern the presentation of health states which severity should be evaluated (e.g., just a disease label or further disease-specific or generic descriptions), the panel of respondents (e.g., experts, the general population or people affected), and the valuation method (common procedures are time trade-off, person trade-off, visual analogue scale, and pairwise comparisons) [12,19]. For the DiWIntox project, recent recommendations [12] and the methodological procedure of the currently largest set of DWs [13,15,20] were considered, accepted, adapted, or rejected regarding the needs of the recent research aim. Consequently, a combination of disease-specific and generic descriptions of moderate and severe CMMVI was used and compared with other health states described in a comparable style (Section 2.1) in an online (Section 2.2) pairwise comparison experiment (Section 2.3). Additionally, a visual analogue scale was used as second valuation method to observe result variation (Section 2.4). The panel of respondents comprised experts (Section 2.5). Data was analyzed with a probit regression in the main analysis (Section 2.6). Details and reasons to all methodological decisions are given in the following.

### 2.1. Health State Descriptions

For DW derivation, a combination of disease-specific and generic health state descriptions is recommended [12]. Corresponding disease descriptions for moderate and severe CMMVI were developed using an expert interview and a literature review [16]. For the generic part of the description, the EuroQol (EQ) questionnaire EQ-5D+C-3L was applied to assess the health-related quality of life. The questionnaire contains the 5 dimensions (5D) mobility, self-care, usual activities, pain/discomfort, and anxiety/depression, as well as an additional dimension, cognition (+C). Each dimension is assessed according to one of three levels (3L) of severity, i.e., the lowest level being “no problems”, the next level “problems”, and the highest level “severe problems”. The EQ-5D+C-3L was chosen because it was used in previous DW studies to describe health states in a generic style [21,22,23,24,25,26,27].

Besides the descriptions of the health states of interest (moderate and severe CMMVI), a set of further health states was needed to compare their disease severity with the severity of CMMVI. The inclusion of further health states was necessary, because the valuation method pairwise comparison was chosen (see Section 2.3), in which the respondents compare two hypothetical individuals with particular health states and decide who is healthier. The requirements regarding the set of other health states were that disease-specific and generic (EQ-5D+C-3L) descriptions are available and that the set contains health states representing the whole spectrum of severities (diseases from low to high severity). Possible descriptions available from previous studies [21,22,23,24,25,26,27] were compared regarding style and length. These assessments entered into the decision whether to include a study in the DiWIntox project.

Finally, 18 health state descriptions from three sources were included. Twelve descriptions, representing a wide range from mild to severe health states, were taken from the European DW project [24]. Three descriptions of disorders attributable to alcoholism were taken from the Dutch DW project [26]. Alcohol use disorders were included to examine the assumption of previous research, expecting that the DW for chronic mercury intoxication might be comparable to the DW of alcoholism [4]. The Dutch study additionally provided the health state description of deafness [26], which was used as an anchor in a visual analogue scale (VAS, see Section 2.4) within a scenario analysis. The decision to include the DW of deafness as an anchor was based on the fact that the DW (0.215) derived by GBD 2013 [15] is in a similar dimension like the DW (0.23) derived or applied in previous studies [26,28,29]. This consistency of the DW derived in different contexts was the reason of its application as an anchor in DiWIntox. Finally, the two descriptions of moderate and severe CMMVI were taken from Steckling et al. [16] and adapted to enhance comparability with the other descriptions. The final health states descriptions (of moderate and severe CMMVI and the other 16 health states) are available in Table S1 of the Supplementary Materials.

In the GBD 2013 study, lay descriptions of the symptoms and functional consequences of the health states developed by expert groups were formulated to enable their accessibility by the general population [13,15,20]. Using the GBD lay descriptions in DiWIntox—which would lead to direct comparability—was no option. One reason was that the target group of DiWIntox contained experts rather than the general population (see Section 2.5). Another reason was that the moderate and severe CMMVI, as previously defined in Steckling et al. [16] are not generally known and accepted disease descriptions. Thus, providing disease-specific and generic descriptions, as recommended [12], was decided to be the best option. However, to check agreements and differences, health state descriptions used in DiWIntox and GBD 2013 were compared by one of the authors, documented in Table S1 (Supplementary Materials), and checked by all co-authors. First, the list of GBD health states was examined to identify GBD health states generally comparable to the DiWIntox health states. The next step was to identify and extract comparable and not comparable parts of the descriptions. Based on this, comparability was categorized as “low”, “medium”, or “high” (Table S1, Supplementary Materials). The comparison was necessary because it was decided to use these DWs of GBD health states to anchor the DiWIntox DWs in the main analysis, which are moderately or highly comparable with the DiWIntox health states (see Section 2.6). Due to this anchoring, the development of DWs within the GBD dimensions was enhanced. 

### 2.2. Data Collection

The online survey tool Unipark (http://www.unipark.com/) was used for data collection. A pretest was conducted from 2 March to 7 March 2016 to test the comprehension and quality of the web survey. Feedback from the 24 pretest respondents and lessons learned during the pretest were taken into account and the final version of the online questionnaire was opened for participation from 27 March to 4 April 2016. The invitation was sent by email to the experts identified (see Section 2.5) with a request to invite further colleagues from their own research field to participate in the survey by forwarding the email. 

A hyperlink opened the welcome letter and the anonymous survey started when the respondents gave their informed consent to participate. Content of the survey were pairwise comparison (PC) questions and a visual analogue scale (VAS) exercise for which answers were obligatory to continue the survey. Further questions regarding the respondents’ expertise and socioeconomic characteristics were optional. The survey ended with a free text entry field for comments and a thank you letter (Table S2, Supplementary Materials). 

The questionnaire was available in English. Concerns regarding data protection were discussed with a data protection officer. An ethical approval was obtained from the ethical committee of Bielefeld University (ethic project code: 2016-033). 

### 2.3. Pairwise Comparison (PC)

The respondents were asked to compare a person suffering from a defined health state with a person suffering from another defined health state, and to decide who is healthier overall. Indifference was not available as an option. Comparing all of the 18 health states with all other health states would result in 18 × 17 = 306 possible combinations; a number too high for a single respondent to answer. A feasible questionnaire was created including 10 PC questions per respondent. The health states were presented using the health state descriptions selected (see Section 2.1).

Participants were randomly assigned to answer one out of seven different sets of 10 PC questions each. The seven sets differed in the pairs of health states they contained. The pairs as well as the position of the health states (first or second option) within a PC question were randomly assigned. The sequence of PC questions within a set was assigned randomly to each participant.

Every health state was presented to every respondent who finalized the questionnaire completely at least once and at most twice. For each participant, one PC question was repeated in reverse order to check the test-retest reliability as has been done in previous projects [13,15,29]. The repeated question included moderate or severe CMMVI in comparison to any other health state. Table S3 in the Supplementary Materials contains the list of PC questions per set. Figures S2–S17 in the Supplementary Materials contain screenshots of one complete online questionnaire version.

### 2.4. Visual Analogue Scale (VAS)

A Visual Analogue Scale (VAS) was included as an add-on in the survey to test result variation in accordance to an alternative survey method (see Section 2.6, Scenario Analysis 2). The VAS contained values from 0, presenting the worst imaginable health state, to 100, presenting the best imaginable health state. The respondents were asked to indicate the position of one given health state on the scale by selecting a value between 0 and 100 under consideration of a predefined value of 78 for deafness (taking into account the DW for deafness derived by Stouthard et al. [26] and Salomon et al. [15]). The respondents were randomly assigned to evaluate one out of three health states (quadriplegia, moderate or severe CMMVI). Following the intention to determine the value of an upper anchor also represented in other studies, quadriplegia was subject to evaluation in five of seven cases. Additionally, moderate and severe CMMVI were evaluated in one of seven cases each, because these health states were of main interest in this study. 

### 2.5. Panel Composition

Two subgroups of experts were recruited for participation. Experts involved in the health research of mercury were asked to participate. These experts were identified by conducting a PubMed search of health-related mercury articles published in the last five years. The search terms were combinations of two or all of the three of the following aspects: cause (*mercury*), effect (represented by the terms *disease*, *health*, *intoxication*, *poisoning*), setting (*gold mining*). 

While the answers of the mercury experts might be influenced by their research interest, further scientists without special research interest in mercury but with general health-related knowledge were asked to participate. While this broad subgroup definition describes a countless number of possible participants, a specific focus on BoD experts was chosen. These were identified by a PubMed search of the term *disability weight/s* (limitation: last 5 years). The advantages of this strategy are that the participants are interested in DWs and that they know their importance for BoD research. By inviting this subgroup, a higher response rate was assumed than by inviting individuals without or with restricted knowledge about BoD. 

The corresponding authors of the mercury and DW paper identified were invited by email to answer the questionnaire. The invitation (Table S4, Supplementary Materials) included the request to forward the email to further colleagues from their research field. This was done to increase the number of participants. At the end of the survey, participants were requested to reveal their expertise to check whether the group of respondents agreed with the group of invitees.

The study group included experts rather than the general population or affected people. DWs were derived under the assumption of universality/no consideration of country/culture/other circumstances.

### 2.6. Data Management and Analyses

Data obtained by VAS and questions about expertise and socioeconomic information were in a format which did not require specific adaption before analyses. Descriptive statistics were performed in SPSS (IBM Corporation, Armonk, NY, USA). Data obtained by the PC questions needed adaption before analyses. The answers to the PC questions collected by the online questionnaire tool were stored in 70 PC variables. Three values were possible: 1, the respondent answered that the person with the first health state is healthier; 2, the respondent answered that the person with the second health state is healthier; −99, the respondent did not answer the question by closing the survey before finalization or the question was not presented to the respondent (missing value). Data were stored in a matrix with one row per participant and one column per PC question.

To analyze the data with probit regression, the matrix had to be changed in one column containing the results of all PC questions and several rows per participant (e.g., row 1: answer of participant 1 to PC 1; row 2: answer of participant 1 to PC 2, and so on). Outgoing from this, further necessary variables were generated (Table 1).

The results of the PC questions were analyzed using probit regression [13,15,20]. The binary dependent variable contained 1 when the first health state was chosen as healthier in the PC question and 0 otherwise. Independent variables were generated for every health state containing 1 when the health state was the first option in the pair, −1 when the health state was the second option in the pair, and 0 when the respondent was not asked to answer the PC question or when the respondent closed the survey without answering the PC question. For quality assurance, the probit regression was independently run by two researchers using the statistic program R (R Core Team; Vienna, Austria) and STATA (StataCorp LLC; College Station, TX, USA) respectively.

The probit regression coefficients were translated into DWs by the application of four approaches (one main analysis and three scenario analyses, Table 2). The main analysis is based on the most sophisticated approach. Scenario analyses were done to observe result variation by applying alternative survey and data analyses approaches.

#### 2.6.1. Main Analysis

In the main analysis, local regression (LOESS) was used to translate regression coefficients into DWs. The dependent variable contained the GBD 2013 DWs of the health states, with descriptions that are moderately or highly comparable to the descriptions used in DiWIntox (see Section 2.1), while the independent variable contained the probit regression coefficients of the 18 DiWIntox health states. The scale of the dependent variable was logit transformed to ensure fitted values would fall, after back-transformation, within the 0 to 1 range. A bandwidth of 1 was used, which means that 100% of data were used in smoothing each point. In addition to the observed data points, two theoretical data points were added to anchor the LOESS fit. The values 0.0001 and 0.9999 were added to the dependent variable to limit the 0 to 1 scale. The scale of the independent variable is infinity; this was expressed by including the values 9999 and −9999 (Table 1). 

Based on the fitted LOESS model, predictions, with corresponding standard errors, were made for the probit coefficients, including those for which no GBD 2013 DW was available. An inverse logistic function was used to back-transform these fitted values to the 0 to 1 scale. To propagate the uncertainty resulting from the LOESS fitting process, 1,000,000 Monte Carlo simulations were generated from a normal distribution with mean and standard deviation corresponding to the LOESS estimate and standard error, respectively. After applying the aforementioned back-transformation, the resulting uncertainty distribution was summarized by its mean and a 95% uncertainty interval (UI) defined as the distribution’s 2.5th and 97.5th percentile. 

The DiWIntox DWs of the main analysis were compared with the corresponding GBD DWs. The Spearman’s rank correlation coefficient (Spearman’s Rho) was applied to the mean DiWIntox and GBD DWs to describe the correlation of these variables. 

#### 2.6.2. Scenario Analysis 1

Two anchor DWs (deafness: 0.215; quadriplegia: 0.589, both taken from GBD 2013) and their probit coefficients were defined as 100% and 0%. For every regression coefficient of the non-anchor health states, the resulting percentage was determined using linear interpolation. Tables S5 and S6 in the Supplementary Materials includes further explanations to convey the fundamental idea behind the data analyses of PC questions. 

#### 2.6.3. Scenario Analysis 2 

The results of the VAS regarding moderate and severe CMMVI, as well as quadriplegia were used in Scenario Analysis 2. VAS data were translated into DWs by applying the following formula:

(1) DW=1−(VAS value/100)

The values were tested regarding meaningfulness. Criteria for exclusion of not meaningful values were developed and applied (see Table S7, Supplementary Materials). Minimum, maximum, mean, and median VAS values with and without exclusion of values were analyzed.

#### 2.6.4. Scenario Analysis 3 

Scenario Analysis 3 used the same approach as applied in Scenario Analysis 1, except that the DW of quadriplegia was taken from the VAS in Scenario Analysis 2 and included as an upper DW anchor. Thus, Scenario Analysis 3 is most widely independent of GBD with the exception that the DW of deafness, which was used as a lower anchor, was predefined based on GBD [15,20] and previous studies [26,28,29,30].

## 3. Results

### 3.1. Descriptive Statistics

On the basis of the PubMed searches, 211 corresponding authors were invited to take part in the survey. People were invited from all over the world, especially from Europe (29%), Asia (25%), and North America (25%). Fewer people were invited from South America (9%), Australia/Oceania (7%), and Africa (5%), what is in accordance to the lower number of publications with the selected keywords from these continents.

A number of 138 participants agreed to take part in the survey and 105 of them completed the entire questionnaire. Among these 105 participants, 60% were male, all were adult, most between 30 and 39 years of age (30%), and 75% were from Europe. For 80% of the participants English was not the native language (Table 3). 

Expertise in public health (60% of participants), epidemiology (50%), and BoD (30%) were most frequently mentioned. Six percent described themselves as mercury experts, while 10% affirmed that they had ever seen anyone suffering from mercury intoxication. Eighty percent of the participants were scientists, 11% were medical doctors, 3% were policy-makers, or had other areas of expertise (Table 3; multiple answers were possible). 

One hundred and seven participants answered one question twice in reverse order of health states. The test-retest reliability reached a value of 86%, representing the interviewees who gave the same answer to both questions. 

### 3.2. Comparability of Health State Descriptions Used in DiWIntox and GBD

The descriptions of the 18 health states included in DiWIntox were used to identify comparable health states used in GBD 2013. For three of the health states in DiWIntox, there are no comparable health states in GBD 2013 (moderate CMMVI; severe CMMVI; diabetes). Comparability of three further health states was evaluated as low (the selected disease stages of breast cancer, colorectal cancer, and HIV/AIDS).

Moderately comparable are the descriptions for chronic low back pain, deafness, delirium and mild vision disorder. The remaining eight health state descriptions show high comparability (Table 4). The GBD DWs of the 11 health states with moderately and highly comparable descriptions to the DiWIntox descriptions were included in the LOESS fit of the main analysis.

### 3.3. Disability Weights

Results of the PC questions were analyzed in the main analysis (Section 3.3.1) as well as in Scenario Analysis 1 (Section 3.3.2). In Scenario Analysis 2 (Section 3.3.3), the VAS results were analyzed. Scenario Analysis 3 (Section 3.3.4) used both, results from PC and VAS (Table 2). Consequently, the following findings were used as starting point in the main analysis and in Scenario Analysis 1 and 3: In total, 1005 PC questions were answered. According to the probit regression, a person with mild vision disorder is healthier than a person with any of the other 17 health states. Of all 18 health states included, severe CMMVI was evaluated as the worst health state following the results of the probit regression. Table S6 in the Supplementary Materials shows the complete order of health states and corresponding probit regression coefficients. These coefficients were transformed into DWs using different approaches in the main analysis (Section 3.3.1) and Scenario Analysis 1 and 3 (Section 3.3.2 and Section 3.3.4). Scenario Analysis 2 (Section 3.3.3) used the VAS results and not the coefficients to derive DWs and additionally provided input to Scenario Analysis 3 (Section 3.3.4). 

#### 3.3.1. Main Analysis 

The main analysis resulted in a DW of 0.368 (95% UI: 0.261–0.484) for the moderate case of CMMVI and a DW of 0.588 (95% UI: 0.193–0.907) for the severe case. Figure S1 in the Supplementary Materials shows the fitted LOESS curve. The dots represent the 11 health states for which moderately and highly comparable GBD health states are available (Table 4). Table 4 also contains the DWs and UI for all 18 health states considered. 

A comparison of the DiWIntox and GBD DWs resulted in the following findings: For the 15 health states for which DiWIntox and GBD DWs were available, the DiWIntox and GBD UIs of 12 health states were overlapping. An interesting finding is the following trend: If the GBD and DiWIntox health state descriptions were highly comparable (*n* = 8), then the DiWIntox and GBD DWs were within each other UI (exception: Coronary heart disease). If the comparability of the health state descriptions was assessed as moderate (*n* = 3), then the DiWIntox and GBD UIs were overlapping, but the DWs were not within each other UI. If a low comparability of health state descriptions was identified (*n* = 4), then the trend was not clear, while in 2 cases the UIs were not overlapping and in the 2 other cases, the DiWIntox and GBD DWs were even within each other UI (see last column in Table 4). The correlation can be described as sufficient by a Spearman’s Rho of 0.77 (*p*-value < 0.001). 

#### 3.3.2. Scenario Analysis 1

In this analysis, linear interpolation of the probit coefficients based on the pairwise comparisons was done, using the same GBD anchor as in the main analysis. Scenario Analysis 2 resulted in a DW range of 0.119 for mild vision disorder to 0.664 for severe CMMVI. The DW for moderate CMMVI was 0.417 (Table 4).

#### 3.3.3. Scenario Analysis 2

Besides the results of the PC questions used in the Main Analysis and Scenario Analysis 1 and 3, Scenario Analysis 2 used the VAS results what resulted in DWs for not more than 3 health states (instead of 18). The integration of moderate and severe CMMVI as well as quadriplegia on a VAS resulted in a mixed picture. The respondents attributed a wide range of values to each of the three health states, resulting in DWs ranging from 0.10 to 1.00 for quadriplegia, 0.15 to 0.93 for severe CMMVI, and 0.00 to 0.80 for moderate CMMVI. The wide range of values determined supports the assumption that some participants had misunderstood the question. Consequently, it was necessary to identify and exclude not meaningful values. These outliers were defined as values which are obviously wrong; namely values higher than the predefined value for deafness (VAS value: 0.78). Based on this rule, 6 values were excluded for quadriplegia, 3 for moderate CMMVI, and one value for severe CMMVI. The median after exclusion of obviously wrong values resulted in a DW of 0.82 for quadriplegia, 0.80 for severe CMMVI, and 0.40 for moderate CMMVI (Table S7, Supplementary Materials). The order of quadriplegia and severe CMMVI is converse in comparison to the results of the main analysis.

#### 3.3.4. Scenario Analysis 3 

Scenario Analysis 3 used the same approach like number 1 with the difference that the VAS DW of quadriplegia derived in Scenario Analysis 2 (see Section 3.3.3) was used as upper anchor instead of the GBD DW. The analysis resulted in a DW range of 0.060 for mild vision disorder to 0.941 for severe CMMVI. The DW for moderate CMMVI was 0.542. The DWs of all 18 health states are presented in Table 4.

## 4. Discussion

DiWIntox is the first project that has derived DWs for mercury intoxication resulting in 0.368 (UI: 0.261–0.484) for moderate and 0.588 (UI: 0.193–0.907) for severe CMMVI in the main analysis. DWs of further 16 health states were presented (Table 4). Previous studies aiming on estimating the DALYs caused by the use of mercury in ASGM were limited due to the missing DW [4,5]. Others focused on renal toxicity due to inorganic mercury [31] or mild mental retardation due to methylmercury [32] including but not limited to ASGM. The results of the current study improve the data basis of BoD analyses while a more precise estimation of the number of DALYs attributable to the use of mercury in ASGM is possible. Thus, the comparability with other diseases and risk factors is enhanced and may give important insights about the relevance of this neglected public health issue. 

Besides the main analysis, scenario analyses applying alternative survey and data analyses approaches were presented. The DWs derived in the main analysis were made comparable to the GBD 2013 DWs [15]. A significant and sufficient Spearman’s Rho was determined, indicating a positive correlation between the DiWIntox and GBD DWs. Thus, the general dimension of the DiWIntox DWs is comparable with those of the largest BoD study available, although methodological differences and restrictions were not avoidable. Meant are especially the use of different health state descriptions and the distinctly smaller number of survey participants in DiWIntox in comparison to GBD.

The DWs derived in scenario analyses with alternative survey and data analyses approaches provided information about methodological aspects and corresponding result variation as well as results (more) independent of GBD 2013.

The order of health states according to their severity in terms of DWs, as derived in the current analyses, is predominately comprehensible. However, there is one inconsistent result depending on the survey instrument (VAS vs. PCs). The VAS resulted in DWs for quadriplegia and severe CMMVI are close with a slightly higher DW for quadriplegia. PCs resulted in a distinct higher DW for severe CMMVI in comparison to quadriplegia. A higher severity of severe CMMVI in comparison to quadriplegia was initially not assumed; however, when comparing the presented EQ-5D+C-3L categorizations, with 333221 for quadriplegia [24] and 233333 for severe CMMVI [16], the result is reasonable. 

It would have been beneficial to have a health state included in this survey with a higher severity than severe CMMVI. This was—according to the high severities in the GBD 2013 study [33]—assumed for severe depression and quadriplegia. The fact that severe CMMVI was the health state on the upper end of the derived DWs might have contributed to the large UI. 

When comparing the order of moderately or highly comparable health state descriptions derived in DiWIntox and GBD 2013 according to the DW value, the following discrepancies arise: the DiWIntox analyses result in a comparable severity of problems of alcohol drinking and severe asthma; GBD 2013 presents a higher DW for the first mentioned. The order of mild dementia and manifest alcoholism is different; however the GBD 2013 DWs of these health states are ranked similarly. The health states coronary heart disease and stroke (moderate impairments) were evaluated as less severe in GBD 2013 than in DiWIntox. Severe depression reached a higher DW than quadriplegia in GBD 2013, although close to each other in ranking. In contrast, the interviewees in the DiWIntox project judged quadriplegia to be more severe than severe depression. As mentioned before, comparability is limited by different health state descriptions. Additionally, in the GBD 2013 study, a large sample size of more than 60,000 interviewees of the general world population were included [33], while in DiWIntox, a small sample of little more than 100 experts took part. Furthermore, some methodological adaptions of the GBD 2013 design were necessary, e.g., population health equivalence questions were not applied in DiWIntox.

In a preliminary estimation of the DALYs attributable to chronic mercury intoxication in Zimbabwe [4], a provisional DW of 0.18 was assumed because a specific DW was not available. The DW was taken from the GBD 1990 study describing the severity of alcohol dependence [8]. The DW of this health state was chosen, because the cause of alcohol dependence is also a substance, the health state is chronic, and similar neuropsychological symptoms are observed as in chronic mercury intoxication [4]. Due to methodological changes during data collection, different health state descriptions, and another group of interviewees, the DWs of the old and recent GBD study are not comparable. However, the congruent health state of alcohol dependence in the DiWIntox project is manifest alcoholism (Table S1, Supplementary Materials). The DW of manifest alcoholism (DW: 0.312) is very close to but below the DW of moderate CMMVI (DW: 0.368). Thus, the previous assumption, that the severity of chronic mercury intoxication is comparable to the severity of alcohol dependence is confirmed as reasonable.

In the DiWIntox project, respondents were recruited by identifying relevant publications and inviting the corresponding authors with the request to forward the invitation to colleagues. The final group of respondents agrees partially with the group of invitees. The corresponding authors of papers about mercury and DW research were invited, however, not more than 6% and less than 30% of respondents identified themselves as a mercury or DW expert, respectively. Due to the recruitment strategy applied, especially public health experts (*n* = 59) and epidemiologists (*n* = 54) were reached. Only a few participants (*n* = 6) confirmed that they had expertise regarding mercury and 10% stated that they saw someone who was mercury intoxicated. Mercury experts could be influenced by their research interest in mercury which could result in a specific response behavior. It is possible that scientists involved in mercury research perceive a higher disease severity of health effects due to mercury than those not involved. To control such effects, the survey was opened for further experts without involvement in mercury research. While finally just a few participants described themselves as a mercury expert, this possible source of bias is not assumable. Consequently, a stratified analysis with the focus on mercury experts seemed not useful because of the restricted number of participants in this subgroup. This point should again be taken up in future research, because mercury experts might have a better understanding of mercury attributable health effects which could imply an enhanced ability to evaluate the severity of such effects. 

The 211 people directly invited to take part in the survey, as identified by PubMed searches, came mostly from Europe (29%), closely followed by North America (25%), and Asia (25%). This order is repeated in the final sample of participants; however, European interviewees are overrepresented with 75% (North America: 15%; Asia: 5%). A reason might be that the invitation to the survey was sent out from Europe. It can be assumed, that the research institution that sent the questionnaire, is better known by the European colleagues, which might have convinced them to participate or to forward the invitation to colleagues. Due to the request to invite colleagues, the total number of people who received the invitation is not known. Besides the small number of invitations sent out to South America (*n* = 20), Australia/Oceania (*n* = 14), and Africa (*n* = 11), a reason for non-participation could be a language barrier for South America and some parts of Africa, because the questionnaire was exclusively available in English. This is also relevant for the final group of participants, because 80% of them had a native language other than English. Consequently, responses to the survey questions could be influenced by misunderstandings. 

Within the project, it was possible to include 18 health states with available corresponding descriptions which were mostly comparable. These were used for pairwise comparisons. Seven sets of pairs were provided and randomly assigned to the participants. Based on the project extent and the assumed low number of participants, this magnitude of variation seemed suitable. Three of the 18 health states were used for VAS, while most participants integrated quadriplegia onto the scale (*n* = 74) and just a few integrated moderate (*n* = 24) and severe (*n* = 16) CMMVI. More attention was given to quadriplegia, because this health state was thought to define the upper end of the severity dimension. The VAS value of quadriplegia was applied in Scenario Analysis 3. Asking for the integration of CMMVI was an add-on to collect more information about mercury intoxication. All in all, VAS resulted in a mixed picture with a wide range of answers. The resulting mean and median DWs are higher than the DWs of the main analysis using PC. Higher values obtained by VAS in comparison to other valuation methods have been reported in earlier studies [12,23,34,35]. 

In summary, the project results are limited by the low number of diseases and the restricted variation of possible pairs presented for evaluation. DiWIntox is a small project with a low number of respondents. Some sub-analyses, like the integration of CMMVI into the VAS, are especially restricted by the number of respondents. The recruitment of participants was limited by predefined criteria. This strategy did not result in participation of a sufficient number of mercury experts. Among the respondents, European experts for public health, epidemiology and BoD were highly represented, for most of whom the survey was in a foreign language. As result of the PCs, severe CMMVI was identified as the most severe health state considered in this study, which might be the reason for the large UI. 

The current results improve the data basis of BoD analyses what enhances the comparability of this condition with other diseases and risk factors. With the DWs derived for moderate and severe CMMVI, a more precise estimation of the number of DALYs attributable to the use of mercury in ASGM is possible. Understanding the public health relevance by determining DALYs gives important insights about the relevance and need for burden reduction of this neglected issue. 

Future research should focus on the following aspects to further improve BoD analyses: The disease-specific health states descriptions and EQ-5D+C-3L categorizations as well as the derivation of DWs should be verified by the conduction of surveys including a representative number of health professionals having experience with mercury intoxicated patients or with personally interviewing patients themselves. Further relevant health states such as mild cases of CMMVI, childhood CMMVI, as well as intoxication due to methylated mercury, need to be described (including disease-specific and generic aspects) and evaluated. The prevalence of CMMVI in ASGM and its distribution in disease stages must be surveyed. 

## 5. Conclusions 

The present study is the first to derive DWs of moderate and severe CMMVI to improve the data basis of mercury-related BoD estimates. This is important to estimate the disease burden attributable to the use of mercury in ASGM. The DWs were smoothed into the GBD 2013 DWs to enhance comparability. In accordance with the results of the DiWIntox project, around 37% (UI: 26% to 48%) of a life year should be counted within an estimation of the BoD in terms of DALYs, if the life year is lived with a moderate CMMVI. For severe cases of CMMVI, a very high severity was ascertained (DW: 0.588, UI: 0.193–0.907), even above the severity of quadriplegia and severe depression. Future research should further strengthen the data basis regarding mercury-related health effects in gold mining to improve estimates about its public health relevance.

## Figures and Tables

**Table 1:** List of variables.

Variable Name	Number of These Kind of Variable	Variable Values and Value Labels	Description and Examples
Q*_hs^#^vs^#^	70, one variable for each pairwise comparison	1 person with the first health state is healthier 2 person with the second health state healthier −99 missing value	Input variable (exported from internet survey tool). Variable contains a 1 if the person with the first health state was evaluated as healthier, 2 if the person with the second health state was evaluated as healthier. Missing values: respondent was not asked to answer the PC question or respondent closed the survey without answering the pairwise comparison (PC) question. *Examples: * q01_hs03vs01; possible variable values: 1, 2, −99 q01_hs04vs10; possible variable values: 1, 2, −99 q70_hs09vs03; possible variable values: 1, 2, −99
hs1_q*	70, one variable for each pairwise comparison	1 breast cancer was health state one in the pair 2 chronic low back pain was health state one in the pair … … 18 quadriplegia was health state one in the pair 0 missing value	Variable contains the number of the first health state of the pairwise comparison (PC) question (see Table S1 in the Supplementary Materials for the numbers of health states and the numbers of PC questions). There is no information in this variable, which health state was chosen as healthier. Missing values: respondent was not asked to answer the PC question or respondent closed the survey without answering the PC question. *Examples: * hs1_q01; possible variable values: 3, 0 hs1_q02; possible variable values: 4, 0 hs1_q70; possible variable values: 9, 0
hs2_q*	70, one variable for each pairwise comparison	1 breast cancer was health state two in the pair 2 chronic low back pain was health state two in the pair … … 18 quadriplegia was health state two in the pair 0 missing value	Variable contains the number of the second health state of the PC question (see Table S1 in the Supplementary Materials for the numbers of health states and the numbers of PC questions). There is no information in this variable, which health state was chosen as healthier. Missing value: respondent was not asked to answer the PC question or respondent closed the survey without answering the PC question. *Examples: * hs2_q01; possible variable values: 1, 0 hs2_q02; possible variable values: 10, 0 hs2_q70; possible variable values: 3, 0
healthier_q*	70, one variable for each pairwise comparison	1 breast cancer was chosen as healthier in the pair 2 chronic low back pain was chosen as healthier in the pair … … 18 quadriplegia was chosen as healthier in the pair . missing value	Variable contains the number of the health state chosen as healthier in the PC question (see Table S1 in the Supplementary Materials for the numbers of health states and the numbers of PC questions). Missing value: respondent was not asked to answer the PC question or respondent closed the survey without answering the PC question. *Examples: * healthier_q01; possible variable values: 1, 3, 0 healthier_q02; possible variable values: 4, 10, 0 healthier_q70; possible variable values: 9, 3, 0
dep_var	one variable	1 the first health state was chosen as healthier 0 otherwise	Dependent variable for probit regression. Contains 1 if the first health state was chosen as healthier in the PC question (see Table S1 in the Supplementary Materials for the number of PC questions); contains 0 if the first health state was not chosen as healthier, if the respondent was not asked to answer the PC question, or respondent closed the survey without answering the PC question. *No examples* (just one variable with two possible values): dep_var; possible variable values: 1, 0
indep_var_hs^#^	18 variables	1 health state was the first option in the pair −1 health state was the second option in the pair 0 missing value	Independent variable for probit regression. For each of the health states, contains 1 if the health state was the first option in the pair, −1 if the health state was the second option in the pair, and 0 if respondent was not asked to answer the PC question or if the respondent closed the survey without answering the PC question. There is no information in this variable, which health state was chosen as healthier *Examples:* indep_var_hs01; possible variable values: 1, −1, 0 indep_var_hs02; possible variable values: 1, −1, 0 indep_var_hs18; possible variable values: 1, −1, 0
gbd_2013	One variable	0.0001 lower bound 0.031 mild vision disorder … … NA moderate CMMVI … … 0.589 quadriplegia NA severe CMMVI 0.9999 upper bound	Dependent variable for the local regression (LOESS). Variable contains GBD 2013 DWs of the 11 health states, which descriptions are moderately or highly comparable to the descriptions used in DiWIntox (see Table 2). For the residual 7 health states without moderately or highly comparable descriptions, not available (NA) was included as placeholder. The values 0.0001 and 0.9999 were added to limit the 0 to 1 scale.
coef	One variable	−9999 upper bound 2.803 mild Vision Disorder … … 1.024 moderate CMMVI … … 0.000 quadripleagia −0.448 severe CMMVI −9999 lower bound	Independent variable for LOESS regression. Variable contains the probit regression coefficients of the 18 DiWIntox health states. The scale of the independent variable is infinity; this was expressed by including the values 9999 and −9999.

The symbols * and ^#^ stand for the question and health state number, respectively.

**Table 2:** Approaches to derive disability weights in main analysis and scenario analyses.

Analysis	Survey Instrument	Data Analysis	Anchor
MA	PC	probit regression, LOESS function	eleven GBD DWs
SA 1	PC	probit regression, rule of three formulas (Formula (S1) in Table S5 in the Supplementary Materials, assuming linearity)	two GBD DWs (deafness and quadriplegia)
SA 2	VAS	See Formula (1)	one GBD DW (deafness)
SA 3	PC, VAS	probit regression, rule of three formulas (Formula (S1) in Table S5 in the Supplementary Materials, assuming linearity)	one GBD DW (deafness) and one DiWIntox VAS DW (quadriplegia; taken from SA 2)

Abbreviations: DW, disability weight; GBD, Global Burden of Disease study [15]; LOESS, local regression; MA, main analysis; PC, pairwise comparison; SA, scenario analysis, VAS, visual analogue scale.

**Table 3:** Sociodemographic characteristics of the interviewees.

Characteristic		Number	Percent (*n* = 105 = 100%, Rounded)
Sex	Male	42	40
	Female	63	60
Age	Younger than 21	0	0
	21–29 years	21	20
	30–39 years	33	31
	40–49 years	21	20
	50–59 years	19	18
	60–69 years	10	10
	70 years and older	1	1
Permanent residence	Africa	1	1
	Asia	5	5
	Australia/Oceania	3	3
	Europe	79	75
	North America	16	15
	South America	0	0
	No answer	1	1
Native language	English	23	22
	Not English	82	78
Expertise *	Burden of Disease	29	27.6
	Chemistry	8	7.6
	Epidemiology	54	51.4
	Medicine	10	9.5
	Mercury	6	5.7
	Politics	3	2.9
	Public Health	59	56.2
	Toxicology	6	5.7
	Others	19	18.1
	No expertise	3	2.9
	No answer	1	1.0
Occupation *	Medical doctor	11	10.5
	Policy maker	3	2.9
	Scientist	82	58.6
	Others	16	15.2
	No occupation	1	1.0
	No answer	0	0.0
Seen someone suffering from mercury intoxication	Yes	10	9.5
	No	94	89.5
	No information	1	1.0
Participants Who Opened the Link to the Survey: 140			
Participants Who Agreed to Take Part: 138			
Participants Who Answered the Survey Completely: 105			

* Multiple answers possible.

**Table 4:** Disability Weights (DWs) of main and scenario analyses in comparison with GBD (Global Burden of Disease) 2013 DWs and comparability of health state descriptions.

DiWIntox Ranking	DiWIntox						GBD Ranking	GBD 2013			DiWIntox vs. GBD 2013	
	Health States (HS) Used in DiWIntox (Ordered by Severity)	DWs (MA)	95% UI (MA)	DWs (SA 1)	DWs (SA 2)	DWs (SA 3)		Health State Best Comparable to DiWIntox Health State	DWs	95% UI	Comparability of HS Description ^~^	Comparison of DiWIntox (MA) and GBD Results *
1	Mild Vision Disorder	0.081	0.033–0.162	0.119	/	0.060	1	Distance vision, mild impairment	0.031	0.019–0.049	moderate	Overlapping UIs
2	Deafness	0.136	0.075–0.221	0.215 ^#^	0.215 ^#^	0.215 ^#^	6	Hearing loss, complete	0.215	0.144–0.307	moderate	Overlapping UIs
3	Breast Cancer (Clinically disease-free stage without permanent sequelae)	0.162	0.098–0.246	0.248	/	0.268	2	Mastectomy	0.036	0.020–0.057	low	Not overlapping UIs
4	Problems of Alcohol Drinking	0.189	0.122–0.274	0.277	/	0.316	7	Alcohol use disorder: mild	0.235	0.160–0.327	high	Overlapping UIs; DWs within each other UI
5	Severe Asthma	0.189	0.122–0.273	0.277	/	0.316	4	Asthma, uncontrolled	0.133	0.086–0.192	high	Overlapping UIs; DW within each other UI
6	Chronic Low Back Pain	0.203	0.134–0.288	0.290	/	0.337	10	Low back pain: severe, with leg pain	0.325	0.219–0.446	moderate	Overlapping UIs
7	HIV/AIDS (seropositive, asymptomatic)	0.208	0.139–0.293	0.296	/	0.346	3	HIV/AIDS: receiving antiretroviral treatment	0.078	0.052–0.111	low	Not overlapping UIs
8	Mild Dementia	0.223	0.152–0.309	0.309	/	0.367	12	Dementia: mild	0.377	0.252–0.508	high	Overlapping UIs
9	Diabetes Mellitus (uncomplicated, poorly controlled)	0.254	0.178–0.343	0.336	/	0.411	/	n.a.	n.a.	n.a.	/	
10	Manifest Alcoholism	0.312	0.223–0.413	0.380	/	0.482	11	Alcohol use disorder: moderate	0.373	0.248–0.508	high	Overlapping UIs; DW within each other UI
11	Coronary Heart Disease, Severe Stable Angina	0.347	0.248–0.458	0.404	/	0.521	5	Angina pectoris: severe	0.167	0.110–0.240	high	Not overlapping UIs
12	Chronic Metallic Mercury Vapor Intoxication (moderate case)	0.368	0.261–0.485	0.417	0.40	0.542	/	n.a.	n.a.	n.a.	/	
13	Colorectal Cancer (Stage of diagnosis and primary therapy)	0.368	0.261–0.485	0.418	/	0.543	8	Cancer: diagnosis and primary therapy	0.288	0.193–0.399	low	Overlapping UIs; DW within each other UI
14	Stroke, moderate impairments	0.431	0.300–0.569	0.458	/	0.608	9	Stroke: long-term consequences, moderate plus cognition problems	0.316	0.206–0.437	high	Overlapping UIs; DW within each other UI
15	Severe Depression	0.526	0.334–0.713	0.536	/	0.734	15	Major depressive disorder: severe episode	0.658	0.477–0.807	high	Overlapping UIs; DW within each other UI
16	Delirium caused by excessive alcohol intake	0.537	0.329–0.736	0.549	/	0.755	13	Alcohol use disorder: severe	0.570	0.396–0.732	low	Overlapping UIs; DW within each other UI
17	Quadriplegia	0.560	0.291–0.804	0.589 ^#^	0.82 ^#^	0.82 ^#^	14	Spinal cord lesion at neck: treated	0.589	0.415–0.748	high	Overlapping UIs; DW within each other UI
18	Chronic Metallic Mercury Vapor Intoxication (severe case)	0.588	0.194–0.907	0.664	0.80	0.941	/	n.a.	n.a.	n.a.	/	/

Abbreviations: DiWIntox: Disability Weight for Chronic Mercury Intoxication; GBD: Global Burden of Disease study; HS: health state; MA: main analysis; n.a.: not available; UI: uncertainty interval. *: Spearman’s Rho applied to the mean DiWIntox DWs of the main analysis and the mean GBD 2013 DWs: 0.77 (*p*-value < 0.001). ^#^: Predefined value. ^~^: Comparability of health state descriptions used in DiWIntox and GBD 2013 was checked and documented in Table S1 (Supplementary Materials).

## Supplementary Materials

The following are available online at www.mdpi.com/1660-4601/14/1/57/s1, Figure S1: Graph of the local regression (LOESS) function; Figure S2: Screenshot of page one of one online survey version; Figure S3: Screenshot of page two of one online survey version; Figure S4: Screenshots of page three of one online survey version; Figure S5: Screenshot of page four of one online survey version; Figure S6: Screenshot of page five of one online survey version; Figure S7: Screenshot of page six of one online survey version; Figure S8: Screenshot of page seven of one online survey version; Figure S9: Screenshot of page eight of one online survey version; Figure S10: Screenshot of page nine of one online survey version,; Figure S11: Screenshot of page ten of one online survey version; Figure S12: Screenshot of page eleven of one online survey version; Figure S13: Screenshot of page twelve of one online survey version; Figure S14: Screenshot of page thirteen of one online survey version; Figure S15: Screenshot of page fourteen of one online survey version; Figure S16: Screenshot of page fifteen of one online survey version; Figure S17: Screenshot of page sixteen of one online survey version; Table S1: Comparison of health state descriptions; Table S2: Structure of the online questionnaire; Table S3: Pairwise comparisons of the seven questionnaire versions; Table S4: Email invitation and reminders to answer the online questionnaire; Table S5: Explanation of Scenario Analysis 1 using a simple linear model to derive disability weights outgoing from two anchors; Table S6: Regression coefficients, percentages outgoing from two anchor and resulting disability weights using a simple linear model (according to scenario Analysis 1); Table S7: Results of integrating quadriplegia, moderate chronic metallic mercury vapor intoxication (CMMVI), and severe CMMVI into a visual analogue scale (VAS; 0: worst imaginable health state; 100: best imaginable health state) with deafness (value 78) predefined and applying exclusion criteria for outlier control.
