# Supplementary material for: Disability Weights for Chronic Mercury Intoxication Resulting from Gold Mining Activities: Results from an Online Pairwise Comparisons Survey
Source: Int J Environ Res Public Health. 2017 Jan 10;14(1):57. doi: 10.3390/ijerph14010057 (PMC5295308; doi:10.3390/ijerph14010057)
Supplement: Supplementary file 1 [file ijerph-14-00057-s001.pdf]

Supplementary Materials: Disability Weights for Chronic Mercury Intoxication Resulting from Gold Mining Activities: Results from an Online Pairwise Comparisons Survey

Nadine Steckling, Brecht Devleesschauwer, Julia Winkelnkemper, Florian Fischer, Bret Ericson, Alexander Krämer, Claudia Hornberg, Richard Fuller, Dietrich Plass and Stephan Bose-O'Reilly

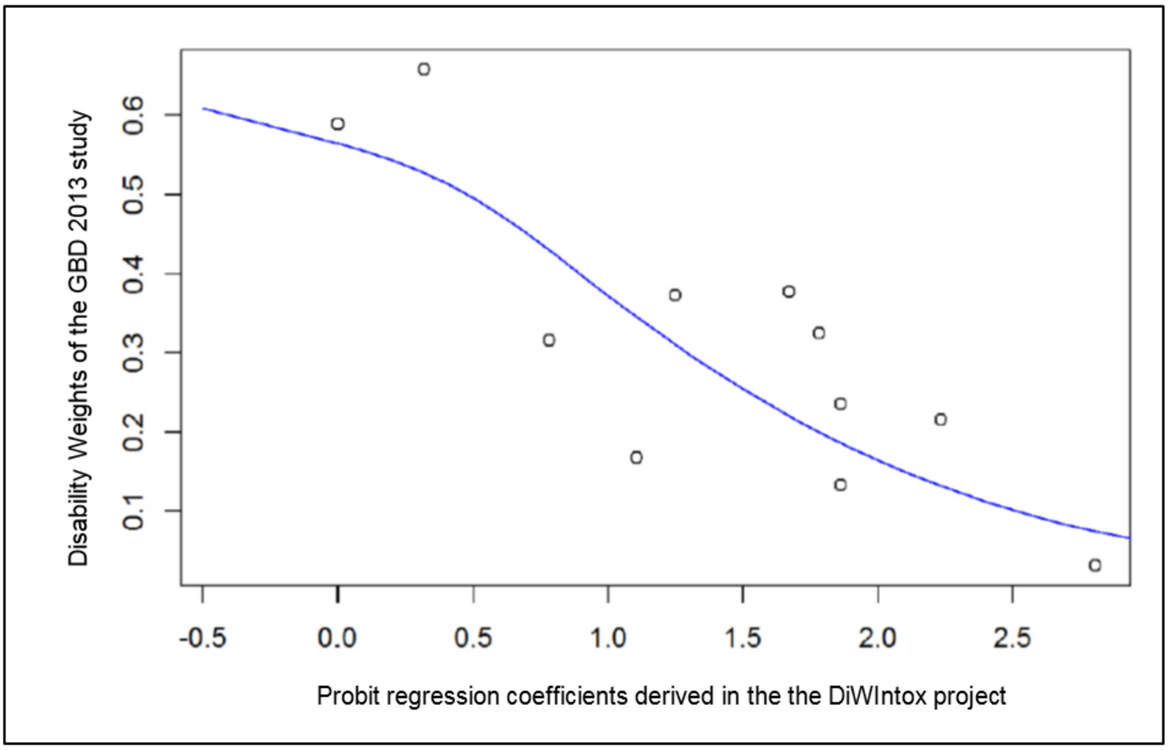

Figure S1. Graph of the LOESS function.

Table S1. Comparison of health state descriptions.

HS #	DiWIntox HS Label (Original Source)	DiWIntox HS Description	Comparable GBD HS Label	GBD 2013 (and 2010) HS Description	Not Comparable Parts of Description (DiWintox vs. GBD)	Comparable Parts of Description (DiWintox—GBD)	Comparability (Low, Medium, High)
1	Breast Cancer (Clinically disease-free stage without permanent sequelae) (Schwarzinger et al., 2003)	Person who has undergone breast conserving therapy and local radiotherapy because of breast cancer more than one year ago, and who only experiences some discomfort; there are no signs of tumour recurrence. EQ-5D+C-3L: 111221	Mastectomy	had one of her breasts removed and sometimes has pain or swelling in the arms.	breast-conserving vs. mastectomy	Breast cancer—cancer	low (both possibilities)
			Cancer: diagnosis and primary therapy	has pain, nausea, fatigue, weight loss and high anxiety.	moderate anxious vs. high anxiety; more than one year ago vs. diagnosis and primary therapy; more/other information vs. less/other information	moderate pain—pain	
2	Chronic low back pain (Schwarzinger et al., 2003)	Person with radiating pain low in the back, limited in sitting and to a smaller extent in walking; patient is generally restricted in all physical activities. EQ-5D+C-3L: 212211	Low back pain: severe, with leg pain	has severe back and leg pain, which causes difficulty dressing, sitting, standing, walking, and lifting things. The person sleeps poorly and feels worried.	Moderate pain vs. severe pain; no problems with dressing vs. difficulty dressing; not anxious vs. feels worried	Radiating pain—with leg pain; some problems in walking—difficulty walking; some problems with performing usual activities—causes difficulty standing and lifting things	moderate
3	Chronic Metallic Mercury Vapor Intoxication (moderate case) (Steckling et al., 2015)	Person with a high level of mercury in his or her body causing slight tremor of fingers, hands, and limb and erethism (psychological disturbances like memory impairment, sleep disorders, shyness, irritability, fatigue). Decreased nerve conduction velocities can be measured. The increased excretion of proteins in urine indicates renal effects. EQ-5D+C-3L: 121222	n.a.	n.a.	/	/	/
4	Chronic Metallic Mercury Vapor Intoxication (severe case) (Steckling et al., 2015)	Person with a very high level of mercury in his or her body causing severe tremor in several parts of the body and severe erethism (psychological disturbances like loss of memory, insomnia, extreme shyness, hyperirritability, fatigue). Person	n.a.	n.a.	/	/	/

		suffers from gingivitis and stomatitis. Paraesthesia, sensory disturbances, reflex abnormalities, and decreased nerve conduction velocities indicate polyneuropathy. A high excretion of proteins in urine indicates abnormal renal functions. EQ-5D+C-3L: 233333					
5	Colorectal Cancer (Stage of diagnosis and primary therapy) (Schwarzinger et al., 2003)	Person after uneventful resection of the colon or bowel after colorectal cancer, still experiencing some pain from the wound; the patient has problems with stool requiring a dietary regimen and medication, and adaptive behaviour in social life. EQ-5D + C-3L: 112221	Cancer: diagnosis and primary therapy	has pain, nausea, fatigue, weight loss and high anxiety.	Moderately anxious vs. high anxiety; more/other information vs. less/other information; colorectal cancer vs. cancer (in general)	Some/moderate pain—pain	Low
6	Coronary heart disease, Severe stable angina (Schwarzinger et al., 2003)	Person with recurrent attacks of severe chest pain, typically provoked by mild exertion (such as walking up a short flight of stairs) or by cold weather. Attacks usually end within minutes with rest and medication. EQ-5D+C-3L: 212321	Angina pectoris: severe	has chest pain that occurs with minimal physical activity, such as walking only a short distance. After a brief rest, the pain goes away. The person avoids most physical activities because of the pain.	more/other information vs. less/other information	provoked by mild exertion—occurs with minimal physical activity; provoked by walking—occurs with walking; Attacks usually end within minutes with rest—after a brief rest, the pain goes away; severe pain~ pain (avoids activity because of pain);	high
7	Deafness (Stouthard et al., 1997; Stouthard et al., 2000)	Person with severe congenital or early acquired hearing disorder. EQ-5D + C-3L: 113111	Hearing loss, complete	GBD 2013: cannot hear at all in any situation, including even the loudest sounds, and cannot communicate verbally or use a phone. Difficulties with communicating and relating to others often cause worry, depression or loneliness. (GBD 2010: cannot hear at all, even loud sounds).	not depressed vs. depression; less/other information vs. more/other information	Severe hearing disorder—cannot hear; some problems with performing usual activities—cannot communicate	moderate

8	Delirium caused by excessive alcohol intake (Stouthard et al., 1997; Stouthard et al., 2000)	Person with psycho-organic disorder (delirium) caused by excessive alcohol intake. EQ-5D + C-3L: 233233	Alcohol use disorder: severe	gets drunk almost every day and is unable to control the urge to drink. Drinking and recovering replace most daily activities. The person has difficulty thinking, remembering and communicating, and feels constant pain and fatigue.	Delirium (psycho-organic disorder) vs. severe alcohol use disorder; excessive alcohol intake vs. gets drunk almost every day; severe problems with performing usual activities vs. drinking and recovering replace most daily activities; moderate pain or discomfort—constant pain and fatigue; severe problems in cognitive functions—has difficulty thinking, remembering and communicating; other information vs. other information	Caused by alcohol—alcohol use disorder	low
9	Diabetes Mellitus (uncomplicated, poorly controlled) (Schwarzinger et al., 2003)	Person with poor glycaemic control despite careful adherence to insulin and diet, resulting in unexpected hypogly-caemic attacks. EQ-5D+C-3L: 122221	n.a.	n.a.	/	/	/
10	HIV/AIDS (Seropositive, asymptomatic) (Schwarzinger et al., 2003)	Healthy person who knows that an incurable disease is acquired. EQ-5D+C-3L: 111121	HIV: symptomatic, pre-AIDS	has weight loss, fatigue, and frequent infections.	asymptomatic vs. symptomatic; AIDS vs. pre-AIDS	HIV—HIV	Low (both possibilities)
			HIV/AIDS: receiving antiretroviral treatment	has occasional fevers and infections. The person takes daily medication that sometimes causes diarrhea.	asymptomatic vs. occasional fevers and infections; other information vs. other information	HIV—HIV	
11	Manifest alcoholism (Stouthard et al., 1997; Stouthard et al., 2000)	Person who has manifest alcoholism. Severe social problems caused by excessive alcohol intake. EQ-5D+C-3L: 113221	Alcohol use disorder: moderate	drinks a lot, gets drunk almost every week and has great difficulty controlling the urge to drink. Drinking and recovering cause great difficulty in daily activities, sleep loss, and fatigue.	more/other information vs. less information	Manifest alcoholism—drinks a lot; unable to perform usual activities—great difficulty in daily activities; severe social problems—great difficulty controlling the urge to drink	high

			Excursion: Health state description used in the GBD 1990 study: Dependence syndrome: Chronic alcoholism (ICD-10: F10.2) A cluster of behavioural, cognitive, and physiological phenomena that develop after repeated substance use and that typically include a strong desire to take the drug, difficulties in controlling its use, persisting in its use despite harmful consequences, a higher priority given to drug use than to other activities and obligations, increased tolerance, and sometimes a physical withdrawal state. The dependence syndrome may be present for a specific psychoactive substance (e.g., tobacco, alcohol, or diazepam), for a class of substances (e.g., opioid drugs), or for a wider range of pharmacologically different psychoactive substances.			
12	Mild Dementia (Schwarzinger et al., 2003)	Person with mild loss of recent memory and some problems in planning and organising daily activities; the person is aware of the deterioration in cognitive functioning; the person is still capable of living independently. EQ-5D+C-3L: 112122	Dementia: mild	has some trouble remembering recent events, and finds it hard to concentrate and make decisions and plans.	more/other information vs. less information	mild loss of recent memory—some trouble remembering recent events; some problems in planning and organising daily activities—finds it hard to make decisions and plans; some problems in cognitive function—finds it hard to concentrate.	high
13	Mild Vision Disorder (Schwarzinger et al., 2003)	Person experiencing some difficulty to read small newspaper print, despite sufficient correction with glasses, and no difficulty in recognising faces at 4 m. distance. EQ-5D+C-3L: 112111	Distance vision, mild impairment	has some difficulty with distance vision, for example reading signs, but no other problems with eyesight.	Farsighted vs. nearsighted; more/other information vs. less information	Vision disorder—vision impairment	medium
14	Problems of alcohol drinking (Stouthard et al., 1997; Stouthard et al., 2000)	Person with problems of alcohol drinking, i.e., some physical, psychological or social problems caused by excessive alcohol intake. EQ-5D+C-3L: 112121	Alcohol use disorder: mild	drinks a lot of alcohol and sometimes has difficulty controlling the urge to drink. While intoxicated, the person has difficulty performing daily activities.	more/other information vs. less/other information	some problems with performing usual activities—difficulty performing daily activities; excessive alcohol intake—drinks a lot of alcohol	high
15	Severe asthma (Schwarzinger et al., 2003)	Person with recurrent attacks of severe shortness of breath, despite adequate therapy and careful adherence to therapy; these attacks limit the daily activities. EQ-5D+C-3L: 112221	Asthma, uncontrolled	has wheezing, cough and shortness of breath more than twice a week, which causes difficulty with daily activities and sometimes wakes the person at night.	other information vs. other information	despite adequate therapy and careful adherence to therapy—uncontrolled; recurrent attacks—more than twice a week; shortness of breath = shortness of breath; limit the daily activities—difficulty with daily activities	high
16	Severe Depression (Schwarzinger et al., 2003)	Person with strong feelings of emptiness and sadness, who has lost all interest, pleasure and energy, and has severe	Major depressive disorder:	has overwhelming, constant sadness and cannot function in daily life. The person sometimes loses touch with	more/other information vs. less/other information	strong feelings of sadness—constant sadness; unable to perform usual activities—cannot function in daily life;	high

		disturbances of sleep, appetite, concentration and cognition, despite treatment; daily activities can not be performed. EQ-5D + C-3L: 223232	severe episode	reality and wants to harm or kill himself (or herself).		extremely anxious or depressed—wants to harm or kill himself (or herself)	
17	Stroke, moderate impairments (Acute incident plus rehabilitation phase) (Schwarzinger et al., 2003)	Person with permanent impairments in movement, speech and memory after incomplete recovery from a stroke more than one year ago. EQ-5D+C-3L: 222222	Stroke: long-term consequences, moderate plus cognition problems	has some difficulty in moving around, in using the hands for lifting and holding things, dressing and grooming, and in speaking. The person is often forgetful and confused	more/other information vs. less/other information	Acute incident plus rehabilitation phase—long-term consequences; stroke more than one year ago—long-term consequences; moderate impairments—moderate problems ; some problems in cognitive functioning—cognition problems/confused; permanent impairments in movement/some problems in walking about—some difficulty in moving around; permanent impairments in speech—some difficulty in speaking; permanent impairments in memory—forgetful; some problems with washing or dressing self—some difficulty in dressing and grooming	high
18	Quadriplegia (Schwarzinger et al., 2003)	Person who is paralyzed from the neck downwards but can breathe independently; he/she is unable to move his/her arms or hands and is confined to a bed or special wheelchair. EQ-5D+C-3L: 333221	Spinal cord lesion at neck: treated	GBD 2013: is paralyzed from the neck down, with no feeling or control over any part of the body below the neck, and no urine or bowel control. (GBD 2010: is paralyzed from the neck down and cannot feel or move the arms and legs.)	more/other information vs. less/other information;	paralysed from the neck downwards—paralyzed from the neck down; is confined to a bed or special wheelchair—with no control over any part of the body below the neck	high

Table S2. Structure of the online questionnaire.

Page	Content	Number of Questions	
		Per Page	Total
1	Welcome letter and informed consent to participate	0	0
2–11 *	Pairwise comparison (random assignment to one out of 7 sets of 10 PC questions **)	1	10
12 *	Visual analog scale (VAS) (random assignment to one out of 3 VAS questions)	1	1
13	Information of expertise	3	3
14	Socioeconomic information	5	5
15	Comments to the survey and thank you letter	1	1
	Total number of questions		20

* The order of these questions was randomly changed for every participant. ** For every respondent, one PC question (containing moderate or severe CMMVI in comparison to another health state) was repeated in reverse order of health states to test the test-retest reliability.

Table S3. Pairwise comparisons of the seven questionnaire versions.

Set	Question (q)	Health State (HS) vs. HS	No. of HS	
			1st	2nd
1	q01	Chronic Metallic Mercury Vapor Intoxication (moderate case) vs. Breast Cancer (Clinically disease-free stage without permanent sequelae)	3	1
	q02	Chronic Metallic Mercury Vapor Intoxication (severe case) vs. HIV/AIDS (seropositive, asymptomatic)	4	10
	q03	Colorectal Cancer (Stage of diagnosis and primary therapy) vs. Diabetes Mellitus (uncomplicated, poorly controlled)	5	9
	q04	Coronary Heart Disease, Severe Stable Angina vs. Deafness	6	8
	q05	Severe Depression vs. Problems of Alcohol Drinking	16	14
	q06	Mild Dementia vs. Chronic Low Back Pain	12	2
	q07	Manifest Alcoholism vs. Mild Vision Disorder	11	13
	q08	Stroke, moderate impairments vs. Delirium caused by excessive alcohol intake	17	7
	q09	Severe Asthma vs. Quadriplegia	15	18
	q10	**Retest Question:** Breast Cancer (Clinically disease-free stage without permanent sequelae) vs. Chronic Metallic Mercury Vapor Intoxication (moderate case)	1	3
2	q11	Chronic Metallic Mercury Vapor Intoxication (moderate case) vs. Problems of Alcohol Drinking	3	14
	q12	Chronic Metallic Mercury Vapor Intoxication (severe case) vs. Colorectal Cancer (Stage of diagnosis and primary therapy)	4	5
	q13	HIV/AIDS (seropositive, asymptomatic) vs. Delirium caused by excessive alcohol intake	10	7
	q14	Deafness vs. Mild Vision Disorder	8	13
	q15	Severe Depression vs. Manifest Alcoholism	16	11
	q16	Mild Dementia vs. Deafness	12	8
	q17	Breast Cancer (Clinically disease-free stage without permanent sequelae) vs. Quadriplegia	1	18
	q18	Stroke, moderate impairments vs. Diabetes Mellitus (uncomplicated, poorly controlled)	17	9
	q19	Severe Asthma vs. Chronic Low Back Pain	15	2
	q20	**Retest Question:** Problems of Alcohol Drinking vs. Chronic Metallic Mercury Vapor Intoxication (moderate case)	14	3
3	q21	Chronic Metallic Mercury Vapor Intoxication (moderate case) vs. Coronary Heart Disease, Severe Stable Angina	3	6
	q22	Chronic Metallic Mercury Vapor Intoxication (severe case) vs. Stroke, moderate impairments	4	17

	q23	HIV/AIDS (seropositive, asymptomatic) vs. Chronic Low Back Pain	10	2
	q24	Manifest Alcoholism vs. Colorectal Cancer (Stage of diagnosis and primary therapy)	11	5
	q25	Severe Depression vs. Severe Asthma	16	15
	q26	Mild Dementia vs. Diabetes Mellitus (uncomplicated, poorly controlled)	12	9
	q27	Breast Cancer (Clinically disease-free stage without permanent sequelae) vs. Delirium caused by excessive alcohol intake	1	7
	q28	Quadriplegia vs. Problems of Alcohol Drinking	18	14
	q29	Coronary Heart Disease, Severe Stable Angina vs. Mild Vision Disorder	6	13
	q30	**Retest Question:** Coronary Heart Disease, Severe Stable Angina vs. Chronic Metallic Mercury Vapor Intoxication (moderate case)	6	3
4	q31	Chronic Metallic Mercury Vapor Intoxication (moderate case) vs. Mild Dementia	3	12
	q32	Chronic Metallic Mercury Vapor Intoxication (severe case) vs. Severe Depression	4	16
	q33	HIV/AIDS (seropositive, asymptomatic) vs. Colorectal Cancer (Stage of diagnosis and primary therapy)	10	5
	q34	Manifest Alcoholism vs. Severe Asthma	11	15
	q35	Diabetes Mellitus (uncomplicated, poorly controlled) vs. Problems of Alcohol Drinking	9	14
	q36	Stroke, moderate impairments vs. Mild Vision Disorder	17	13
	q37	Breast Cancer (Clinically disease-free stage without permanent sequelae) vs. Chronic Low Back Pain	1	2
	q38	Quadriplegia vs. Coronary Heart Disease, Severe Stable Angina	18	6
	q39	Deafness vs. Delirium caused by excessive alcohol intake	8	7
	q40	**Retest Question:** Severe Depression vs. Chronic Metallic Mercury Vapor Intoxication (severe case)	16	4
5	q41	Chronic Metallic Mercury Vapor Intoxication (moderate case) vs. Deafness	3	8
	q42	Chronic Metallic Mercury Vapor Intoxication (severe case) vs. Severe Asthma	4	15
	q43	HIV/AIDS (seropositive, asymptomatic) vs. Breast Cancer (Clinically disease-free stage without permanent sequelae)	10	1
	q44	Manifest Alcoholism vs. Stroke, moderate impairments	11	17
	q45	Diabetes Mellitus (uncomplicated, poorly controlled) vs. Chronic Low Back Pain	9	2
	q46	Colorectal Cancer (Stage of diagnosis and primary therapy) vs. Severe Depression	5	16
	q47	Mild Vision Disorder vs. Mild Dementia	13	12
	q48	Problems of Alcohol Drinking vs. Coronary Heart Disease, Severe Stable Angina	14	6
	q49	Delirium caused by excessive alcohol intake vs. Quadriplegia	7	18
	q50	**Retest Question:** Severe Asthma vs. Chronic Metallic Mercury Vapor Intoxication (severe case)	15	4
6	q51	Chronic Metallic Mercury Vapor Intoxication (moderate case) vs. Mild Vision Disorder	3	13
	q52	Chronic Metallic Mercury Vapor Intoxication (severe case) vs. Chronic Low Back Pain	4	2
	q53	Colorectal Cancer (Stage of diagnosis and primary therapy) vs. Delirium caused by excessive alcohol intake	5	7
	q54	Coronary Heart Disease, Severe Stable Angina vs. Breast Cancer (Clinically disease-free stage without permanent sequelae)	6	1
	q55	Severe Depression vs. HIV/AIDS (seropositive, asymptomatic)	16	10
	q56	Mild Dementia vs. Problems of Alcohol Drinking	12	14
	q57	Manifest Alcoholism vs. Deafness	11	8
	q58	Stroke, moderate impairments vs. Quadriplegia	17	18

	q59	Severe Asthma vs. Diabetes Mellitus (uncomplicated, poorly controlled)	15	9
	q60	**Retest Question:** Mild Vision Disorder vs. Chronic Metallic Mercury Vapor Intoxication (moderate case)	13	3
7	q61	Chronic Metallic Mercury Vapor Intoxication (moderate case) vs. Diabetes Mellitus (uncomplicated, poorly controlled)	3	9
	q62	Chronic Metallic Mercury Vapor Intoxication (severe case) vs. Problems of Alcohol Drinking	4	14
	q63	Colorectal Cancer (Stage of diagnosis and primary therapy) vs. Quadriplegia	5	18
	q64	Coronary Heart Disease, Severe Stable Angina vs. Chronic Low Back Pain	6	2
	q65	Severe Depression vs. Mild Vision Disorder	16	13
	q66	Mild Dementia vs. Breast Cancer (Clinically disease-free stage without permanent sequelae)	12	1
	q67	Manifest Alcoholism vs. HIV/AIDS (seropositive, asymptomatic)	11	10
	q68	Stroke, moderate impairments vs. Deafness	17	8
	q69	Severe Asthma vs. Delirium caused by excessive alcohol intake	15	7
	q70	**Retest Question:** Diabetes Mellitus (uncomplicated, poorly controlled) vs. Chronic Metallic Mercury Vapor Intoxication (moderate case)	1	3

The combination of health states in PC questions as well as their position (first or second option) was randomly assigned for every of the seven sets. The sequence in which the PC questions were presented to the respondents was randomly assigned for every respondent. Example: respondent 1 and 7 answered set 4, respondent 5, 2, and 9 answered set 2 and so on. Respondent 5 answered PC 3 of set 2 as first question.

Table S4. Screenshots of one online survey version.

6%

Dear Sir/Madame,
Welcome to the survey about how people compare different health states.

In the following you are asked a series of questions; each including health states of two different people. You should imagine that these two people experience the described health states for exactly the same time period. Please decide which person you think is healthier overall, in terms of having fewer physical and/or mental limitations on what they can do in life. Some of the questions may be easy to answer, while others may be harder. We are interested in your personal views.

The survey will take about 10 minutes.

Thank you very much for participating.

The project received funding from Pure Earth and Bielefeld University and is a cooperation of the University Hospital Munich, the German Environment Agency, and Bielefeld University. If you wish more information about the survey, please contact Nadine Steckling from the University Hospital Munich, Institute and Outpatient Clinic for Occupational, Social and Environmental Medicine: nadine.steckling@med.uni-muenchen.de.

Statement of agreement
By proceeding with the survey you are consenting to take part in this research project. The survey is anonymous and your IP address will not be saved. There is no possibility to connect answers to participants. You are answering the questions voluntary.

○ I confirm my agreement.

In this survey, some text passages were taken word-by-word or adapted from Rehm&Frick (2013), Salomon et al. (2012), Schwarzinger et al. (2003), Schwarzinger (personal communication), Steckling et al. (2015) and van Reenen & Oppe (2015).

Continue

13%

Who do you think is healthier overall, the first person or the second person?

Imagine that the two people described below will experience the described health states for exactly the same time period. Please decide which person you think is healthier overall, in terms of having fewer physical and/or mental limitations on what they can do in life.

The first person has **Problems of Alcohol Drinking**

Person with problems of alcohol drinking, i.e., some physical, psychological or social problems caused by excessive alcohol intake.

The person
- has no problems in walking about
- has no problems with washing or dressing self
- has some problems with performing usual activities (e.g. work, study, housework, family or leisure activities)
- has no pain or discomfort
- is moderately anxious or depressed
- has no problems in cognitive functioning (e.g. memory, learning ability, concentration, comprehension)

The first person is healthier

The second person has **Chronic Metallic Mercury Vapor Intoxication (moderate case)**

Person with a high level of mercury in his or her body causing slight tremor of fingers, hands, and limb and erethism (psychological disturbances like memory impairment, sleep disorders, shyness, irritability, fatigue). Decreased nerve conduction velocities can be measured. The increased excretion of proteins in urine indicates renal effects.

The person
- has no problems in walking about
- has some problems with self-care
- has no problems with performing usual activities (e.g. work, study, housework, family of leisure activities)
- has moderate pain or discomfort
- is moderately anxious or depressed
- has some problems in cognitive functions (e.g. memory, learning ability, concentration, comprehension)

The second person is healthier

Back

Continue

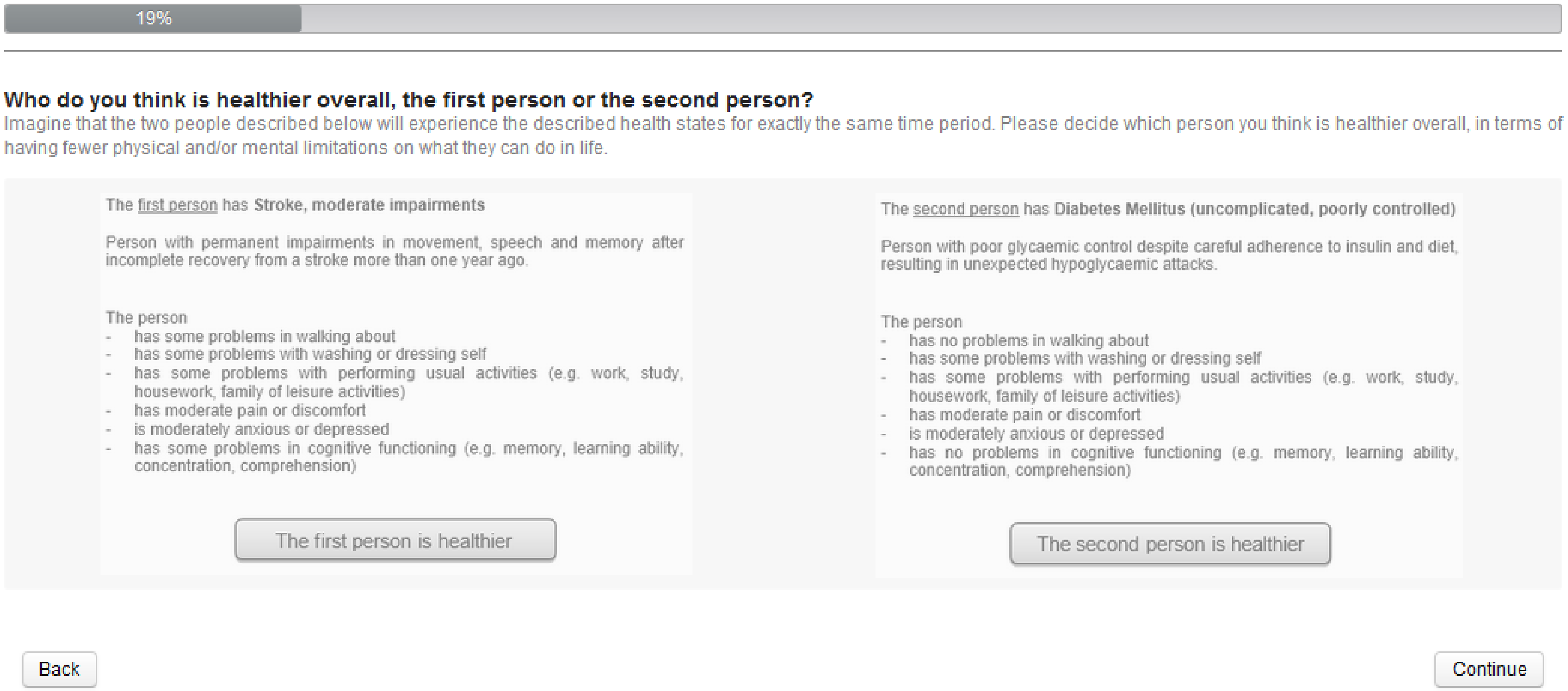

19%

Who do you think is healthier overall, the first person or the second person?

Imagine that the two people described below will experience the described health states for exactly the same time period. Please decide which person you think is healthier overall, in terms of having fewer physical and/or mental limitations on what they can do in life.

The first person has **Stroke, moderate impairments**

Person with permanent impairments in movement, speech and memory after incomplete recovery from a stroke more than one year ago.

The person
- has some problems in walking about
- has some problems with washing or dressing self
- has some problems with performing usual activities (e.g. work, study, housework, family of leisure activities)
- has moderate pain or discomfort
- is moderately anxious or depressed
- has some problems in cognitive functioning (e.g. memory, learning ability, concentration, comprehension)

The first person is healthier

The second person has **Diabetes Mellitus (uncomplicated, poorly controlled)**

Person with poor glycaemic control despite careful adherence to insulin and diet, resulting in unexpected hypoglycaemic attacks.

The person
- has no problems in walking about
- has some problems with washing or dressing self
- has some problems with performing usual activities (e.g. work, study, housework, family of leisure activities)
- has moderate pain or discomfort
- is moderately anxious or depressed
- has no problems in cognitive functioning (e.g. memory, learning ability, concentration, comprehension)

The second person is healthier

Back

Continue

25%

What do you think is the value between 0 and 100 on the scale for Quadriplegia?

Please find on the left side a description of the health state "Quadriplegia" and on the right side a scale ranging from 0, describing the worst imaginable health state, to 100, describing the best imaginable health state. The health state 'Deafness' has a value of 78. Please indicate the position of the health state 'Quadriplegia', in your opinion, by selecting a value between 0 and 100.

In your opinion, the value is: __________

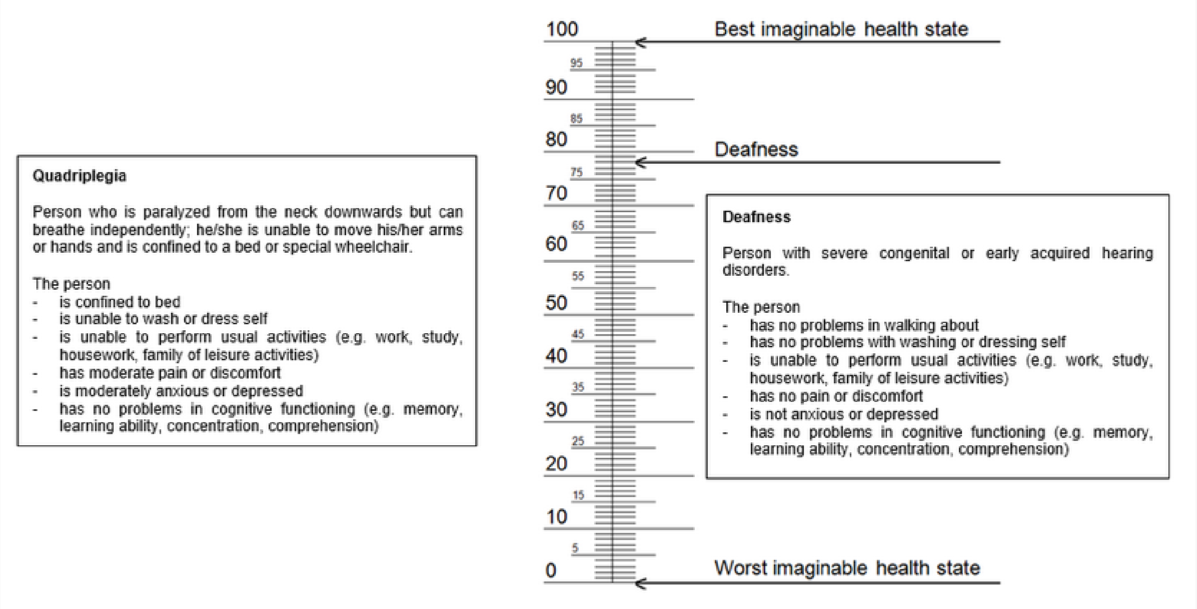

Back Continue

31%

Who do you think is healthier overall, the first person or the second person?

Imagine that the two people described below will experience the described health states for exactly the same time period. Please decide which person you think is healthier overall, in terms of having fewer physical and/or mental limitations on what they can do in life.

The first person has **Mild Dementia**

Person with mild loss of recent memory and some problems in planning and organizing daily activities; the person is aware of the deterioration in cognitive functioning; the person is still capable of living independently.

The person
- has no problems in walking about
- has no problems with washing or dressing self
- has some problems with performing usual activities (e.g. work, study, housework, family of leisure activities)
- has no pain or discomfort
- is moderately anxious or depressed
- has some problems in cognitive functioning (e.g. memory, learning ability, concentration, comprehension)

The first person is healthier

The second person has **Deafness**

Person with severe congenital or early acquired hearing disorders.

The person
- has no problems in walking about
- has no problems with washing or dressing self
- is unable to perform usual activities (e.g. work, study, housework, family of leisure activities)
- has no pain or discomfort
- is not anxious or depressed
- has no problems in cognitive functioning (e.g. memory, learning ability, concentration, comprehension)

The second person is healthier

Back

Continue

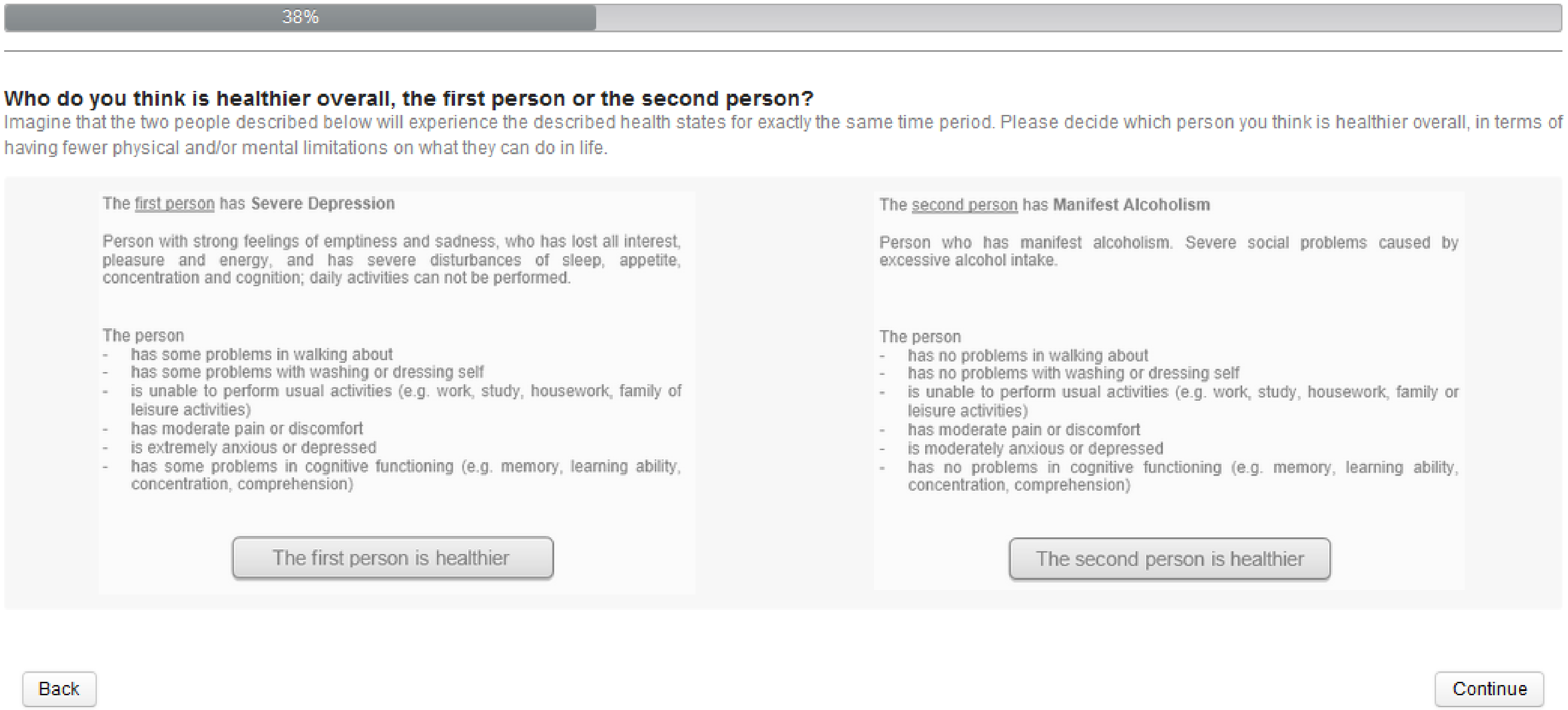

38%

Who do you think is healthier overall, the first person or the second person?

Imagine that the two people described below will experience the described health states for exactly the same time period. Please decide which person you think is healthier overall, in terms of having fewer physical and/or mental limitations on what they can do in life.

The first person has **Severe Depression**

Person with strong feelings of emptiness and sadness, who has lost all interest, pleasure and energy, and has severe disturbances of sleep, appetite, concentration and cognition; daily activities can not be performed.

The person
- has some problems in walking about
- has some problems with washing or dressing self
- is unable to perform usual activities (e.g. work, study, housework, family of leisure activities)
- has moderate pain or discomfort
- is extremely anxious or depressed
- has some problems in cognitive functioning (e.g. memory, learning ability, concentration, comprehension)

The first person is healthier

The second person has **Manifest Alcoholism**

Person who has manifest alcoholism. Severe social problems caused by excessive alcohol intake.

The person
- has no problems in walking about
- has no problems with washing or dressing self
- is unable to perform usual activities (e.g. work, study, housework, family or leisure activities)
- has moderate pain or discomfort
- is moderately anxious or depressed
- has no problems in cognitive functioning (e.g. memory, learning ability, concentration, comprehension)

The second person is healthier

Back

Continue

44%

Who do you think is healthier overall, the first person or the second person?

Imagine that the two people described below will experience the described health states for exactly the same time period. Please decide which person you think is healthier overall, in terms of having fewer physical and/or mental limitations on what they can do in life.

The first person has **Chronic Metallic Mercury Vapor Intoxication (moderate case)**

Person with a high level of mercury in his or her body causing slight tremor of fingers, hands, and limb and erethism (psychological disturbances like memory impairment, sleep disorders, shyness, irritability, fatigue). Decreased nerve conduction velocities can be measured. The increased excretion of proteins in urine indicates renal effects.

The person
- has no problems in walking about
- has some problems with self-care
- has no problems with performing usual activities (e.g. work, study, housework, family or leisure activities)
- has moderate pain or discomfort
- is moderately anxious or depressed
- has some problems in cognitive functions (e.g. memory, learning ability, concentration, comprehension)

The first person is healthier

The second person has **Problems of Alcohol Drinking**

Person with problems of alcohol drinking, i.e., some physical, psychological or social problems caused by excessive alcohol intake.

The person
- has no problems in walking about
- has no problems with washing or dressing self
- has some problems with performing usual activities (e.g. work, study, housework, family or leisure activities)
- has no pain or discomfort
- is moderately anxious or depressed
- has no problems in cognitive functioning (e.g. memory, learning ability, concentration, comprehension)

The second person is healthier

Back

Continue

50%

Who do you think is healthier overall, the first person or the second person?

Imagine that the two people described below will experience the described health states for exactly the same time period. Please decide which person you think is healthier overall, in terms of having fewer physical and/or mental limitations on what they can do in life.

The first person has **HIV / AIDS (seropositive, asymptomatic)**

Healthy person who knows that an incurable disease is acquired.

The person
- has no problems in walking about
- has no problems with washing or dressing self
- has no problems with performing usual activities (e.g. work, study, housework, family of leisure activities)
- has no pain or discomfort
- is moderately anxious or depressed
- has no problems in cognitive functioning (e.g. memory, learning ability, concentration, comprehension)

The first person is healthier

The second person has **Delirium caused by excessive alcohol intake**

Person with psycho-organic disorder (delirium) caused by excessive alcohol intake.

The person
- has some problems in walking about
- is not able to wash or dress themselves
- is not able to perform usual activities (e.g. work, study, housework, family or leisure activities)
- has moderate pain or discomfort
- is extremely anxious or depressed
- has severe problems in cognitive functions (e.g. memory, learning ability, concentration, comprehension)

The second person is healthier

Back

Continue

56%

Who do you think is healthier overall, the first person or the second person?

Imagine that the two people described below will experience the described health states for exactly the same time period. Please decide which person you think is healthier overall, in terms of having fewer physical and/or mental limitations on what they can do in life.

The first person has **Coronary Heart Disease, Severe Stable Angina**

Person with recurrent attacks of severe chest pain, typically provoked by mild exertion (such as walking up a short flight of stairs) or by cold weather. Attacks usually end within minutes with rest and medication.

The person
- has some problems in walking about
- has no problems with washing or dressing self
- has some problems with performing usual activities (e.g. work, study, housework, family of leisure activities)
- has severe pain or discomfort
- is moderately anxious or depressed
- has no problems in cognitive functioning (e.g. memory, learning ability, concentration, comprehension)

The first person is healthier

The second person has **Mild Vision Disorder**

Person experiencing some difficulty to read small newspaper print, despite sufficient correction with glasses, and no difficulty in recognizing faces at 4 m. distance.

The person
- has no problems in walking about
- has no problems with washing or dressing self
- has some problems with performing usual activities (e.g. work, study, housework, family of leisure activities)
- has no pain or discomfort
- is not anxious or depressed
- has no problems in cognitive functioning (e.g. memory, learning ability, concentration, comprehension)

The second person is healthier

Back

Continue

63%

Who do you think is healthier overall, the first person or the second person?

Imagine that the two people described below will experience the described health states for exactly the same time period. Please decide which person you think is healthier overall, in terms of having fewer physical and/or mental limitations on what they can do in life.

The first person has **Breast Cancer (Clinically disease-free stage without permanent sequelae)**

Person who has undergone breast conserving therapy and local radiotherapy because of breast cancer more than one year ago, and who only experiences some discomfort; there are no signs of tumor recurrence.

The person
- has no problems in walking about
- has no problems with washing or dressing self
- has no problems with performing usual activities (e.g. work, study, housework, family of leisure activities)
- has moderate pain or discomfort
- is moderately anxious or depressed
- has no problems in cognitive functioning (e.g. memory, learning ability, concentration, comprehension)

The first person is healthier

The second person has **Quadriplegia**

Person who is paralyzed from the neck downwards but can breathe independently; he/she is unable to move his/her arms or hands and is confined to a bed or special wheelchair.

The person
- is confined to bed
- is unable to wash or dress self
- is unable to perform usual activities (e.g. work, study, housework, family of leisure activities)
- has moderate pain or discomfort
- is moderately anxious or depressed
- has no problems in cognitive functioning (e.g. memory, learning ability, concentration, comprehension)

The second person is healthier

Back

Continue

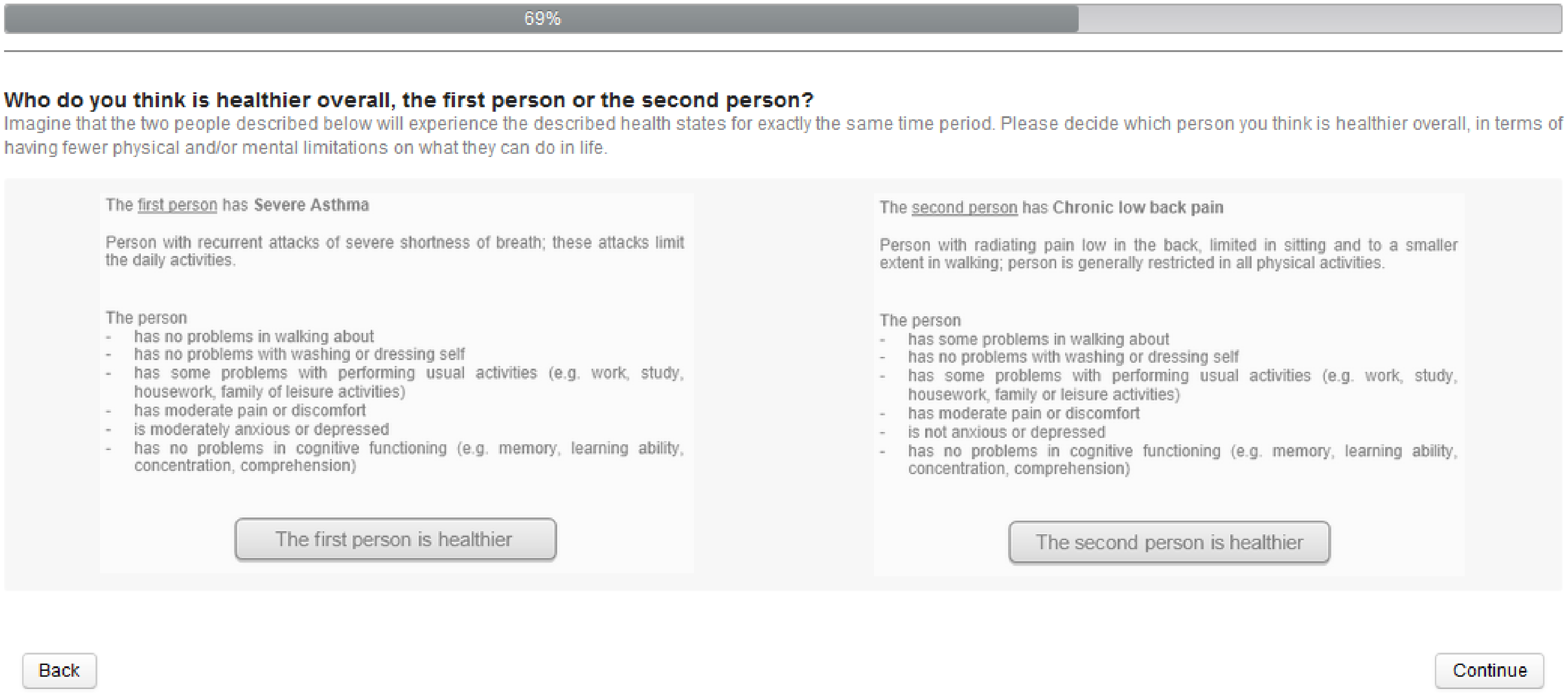

69%

Who do you think is healthier overall, the first person or the second person?

Imagine that the two people described below will experience the described health states for exactly the same time period. Please decide which person you think is healthier overall, in terms of having fewer physical and/or mental limitations on what they can do in life.

The first person has **Severe Asthma**

Person with recurrent attacks of severe shortness of breath; these attacks limit the daily activities.

The person
- has no problems in walking about
- has no problems with washing or dressing self
- has some problems with performing usual activities (e.g. work, study, housework, family of leisure activities)
- has moderate pain or discomfort
- is moderately anxious or depressed
- has no problems in cognitive functioning (e.g. memory, learning ability, concentration, comprehension)

The first person is healthier

The second person has **Chronic low back pain**

Person with radiating pain low in the back, limited in sitting and to a smaller extent in walking; person is generally restricted in all physical activities.

The person
- has some problems in walking about
- has no problems with washing or dressing self
- has some problems with performing usual activities (e.g. work, study, housework, family or leisure activities)
- has moderate pain or discomfort
- is not anxious or depressed
- has no problems in cognitive functioning (e.g. memory, learning ability, concentration, comprehension)

The second person is healthier

Back

Continue

75%

Who do you think is healthier overall, the first person or the second person?

Imagine that the two people described below will experience the described health states for exactly the same time period. Please decide which person you think is healthier overall, in terms of having fewer physical and/or mental limitations on what they can do in life.

The first person has **Chronic Metallic Mercury Vapor Intoxication (severe case)**

Person with a very high level of mercury in his or her body causing severe tremor in several parts of the body and severe erethism (psychological disturbances like loss of memory, insomnia, extreme shyness, hyperirritability, fatigue). Person suffers from gingivitis and stomatitis. Paraesthesia, sensory disturbances, reflex abnormalities, and decreased nerve conduction velocities indicate polyneuropathy. A high excretion of proteins in urine indicates abnormal renal functions.

The person
- has some problems in walking about
- is not able to wash or dress themselves
- is not able to perform usual activities (e.g. work, study, housework, family or leisure activities)
- has severe pain or discomfort
- is extremely anxious or depressed
- has severe problems in cognitive functions (e.g. memory, learning ability, concentration, comprehension)

The first person is healthier

The second person has **Colorectal Cancer (Stage of diagnosis and primary therapy)**

Person after uneventful resection of the colon or bowel after colorectal cancer, still experiencing some pain from the wound; the person has problems with stool requiring a dietary regimen and medication, and adaptive behaviour in social life.

The person
- has no problems in walking about
- has no problems with washing or dressing self
- has some problems with performing usual activities (e.g. work, study, housework, family of leisure activities)
- has moderate pain or discomfort
- is moderately anxious or depressed
- has no problems in cognitive functioning (e.g. memory, learning ability, concentration, comprehension)

The second person is healthier

Back

Continue

81%

What is your occupation? (multiple answers possible)

- ☐ Medical doctor
- ☐ Policy maker
- ☐ Scientist
- ☐ Others:
- ☐ No occupation
- ☐ No information

What is your expertise? (multiple answers possible)

- ☐ Burden of Disease
- ☐ Chemistry
- ☐ Epidemiology
- ☐ Mercury
- ☐ Medicine
- ☐ Politics
- ☐ Public Health
- ☐ Toxicology
- ☐ Others:
- ☐ No expertise
- ☐ No information

Have you ever seen someone suffering from mercury intoxication?

- ○ Yes
- ○ No
- ○ No information

Back Continue

88%

Which age group do you belong to?

- Younger than 21 years
- 21-29 years
- 30-39 years
- 40-49 years
- 50-59 years
- 60-69 years
- 70 years and older
- No information

Are you female or male?

- Female
- Male
- Others
- No information

Where is your permanent residence?

- Africa
- Asia
- Australia/Oceania
- Europe
- North America
- South America
- No information

What is your native language?

- English
- Not English
- No information

Back

Continue

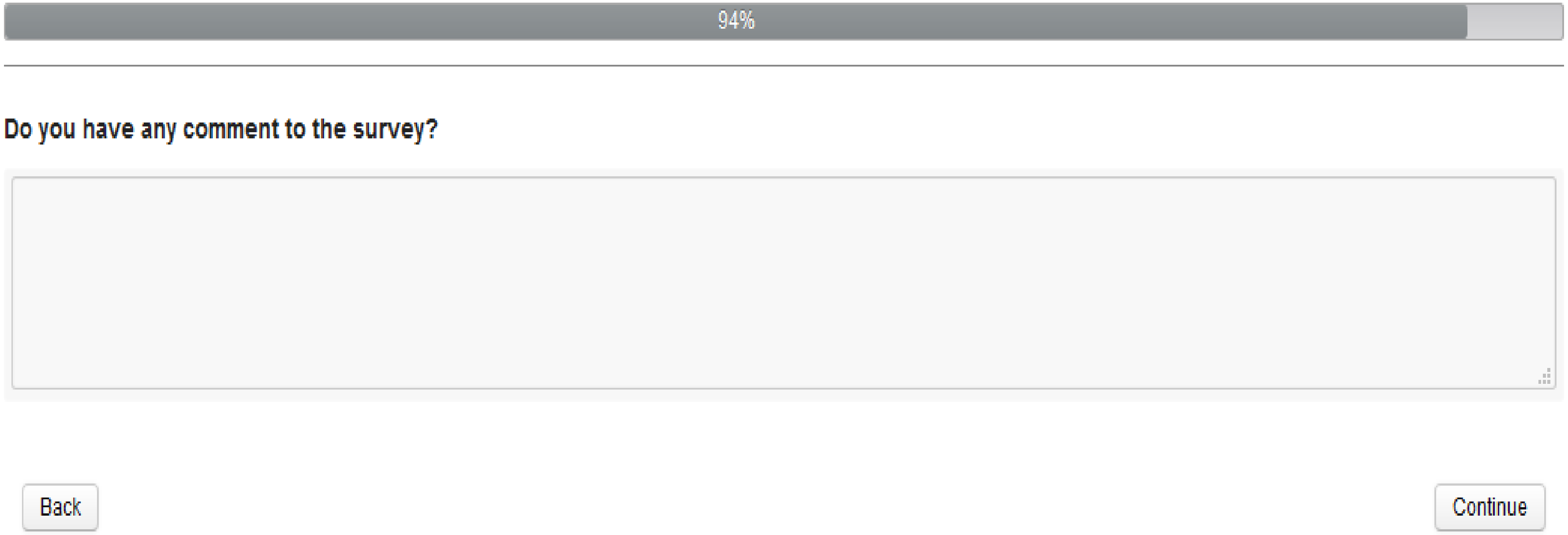
94%
Do you have any comment to the survey?
Back
Continue

100%

Thank you very much for participating!

If you wish more information about the survey, please contact Nadine Steckling from the University Hospital Munich, Institute and Outpatient Clinic for Occupational, Social and Environmental Medicine: nadine.steckling@med.uni-muenchen.de.

The project received funding from Pure Earth and Bielefeld University and is a cooperation of the University Hospital Munich, the German Environment Agency, and Bielefeld University.

Close window

In this survey, some text passages were taken word-by-word or adapted from Rehm&Frick (2013), Salomon et al. (2012), Schwarzinger et al. (2003), Schwarzinger (personal communication), Steckling et al. (2015) and van Reenen & Oppe (2015).

Rehm J, Frick U. Establishing disability weights from pairwise comparisons for a US burden of disease study. Int J Methods Psychiatr Res. 2013 Jun;22(2):144-54.

Salomon J et al. Common values in assessing health outcomes from disease and injury: disability weights measurement study for the Global Burden of Disease Study 2010. Lancet. 2012 Dec 15;380(9859):2129-43.

Schwarzinger M, et al. Cross-national agreement on disability weights: the European Disability Weights Project. Popul Health Metr. 2003 Nov 21;1(1):9.

Steckling N et al. Disease profile and health-related quality of life (HRQoL) using the EuroQol (EQ-5D + C) questionnaire for chronic metallic mercury vapor intoxication. Health Qual Life Outcomes. 2015 Dec 9;13:196.

van Reenen M & Oppe M. EQ-5D-3L User Guide. Basic information on how to use the EQ-5D-3L instrument. Version 5.1. 2015.

Table S5. Email invitation and reminders to answer the DiWIntox-2 questionnaire.

**Invitation**	**Date:** 27/28 March 2016<br>**Subject:** Invitation to participate in a disability weight survey<br><br>Dear colleague,<br><br>Who do you think is healthier: A person with severe asthma or a person with chronic low back pain?<br><br>Interesting question, isn't it? I would like to invite you to take part in a short survey taking about 10 minutes. This survey investigates how people compare different health states. The project received funding from Pure Earth and Bielefeld University and is a cooperation of the University Hospital Munich, the German Environment Agency, and Bielefeld University.<br><br>Please follow this link to start the survey:<br>https://ww2.unipark.de/uc/diwintox_survey/<br><br>We are interested in your opinion. You are receiving this email because you have published at least one PubMed listed paper in the last five years to one of the topics of interest for this survey. Please don't hesitate to invite further colleagues from your research field to this survey by forwarding this email.<br><br>Thank you for supporting our research.<br><br>Best regards<br><br>Nadine<br><br>[*affiliation and contact details*]
**First Reminder**	**Date:** 31 March 2016<br>**Subject:** Friendly reminder: Disability weight survey<br><br>Dear colleague,<br><br>Thanks to all who already participated.<br>If not yet done, please take part in our short survey about how people compare different health states.<br>https://ww2.unipark.de/uc/diwintox_survey/<br>Please don't hesitate to invite further colleagues from your research field to this survey by forwarding this email.<br><br>Thank you for supporting our research.<br>Best regards<br>Nadine<br><br>[*affiliation and contact details*]
**Final Reminder**	**Date:** 3 April 2016<br>**Subject:** Final reminder: please take part in a short survey<br><br>Dear colleagues,

A big thank you to all who already participated in the survey about how people compare different health states. This is the final reminder while the survey will be closed on Monday midnight CET, April 4, 2016.

If not yet done, please take your chance to contribute.
https://ww2.unipark.de/uc/diwintox_survey/

Please don't hesitate to invite further colleagues from your research field to this survey by forwarding this email.
Thank you very much for supporting our research.
Best regards
Nadine

[*affiliation and contact details*]

Table S6. Explanation of scenario analyses 1 using a simple linear model to derive disability weights outgoing from two anchors.

Export regression coefficients to an Excel sheet. Define two anchor health states and extract their DWs from an available study. For DiWIntox-2, the DWs for deafness (0.215) and quadriplegia (0.589) were taken from GBD 2013 (Salomon et al., 2015).The two anchor DWs (deafness: 0.215; quadriplegia: 0.589) and their coefficients as resulted from probit regression were defined as 100% and 0%. For every regression coefficient of the non-anchor health states, the resulting percentage was determined.
Formula (2): $DW_{hsx} = DW_{diff}/100 \times (100 - (100/(coef_{diff}) \times (coef_{hsx} - coef_{quad}))) + DW_{deaf}$<br>$DW_{diff}$: difference between the DW of anchor 1 (deafness) and DW of anchor 2 (quadriplegia)<br>$coef_{diff}$: difference between the coefficient of anchor 1 (deafness) and the coefficient of anchor 2 (quadriplegia)<br>$coef_{hsx}$: coefficient of health state *x*<br>$coeff_{quad}$: coefficient of anchor 2 (quadriplegia)<br>DW_{deaf}: Predefined DW of anchor 1 (deafness)
Example for health state moderate CMMVI:<br>$DW_{modCMMVI} = 0.374/100 \times (100 - (100/(2.232) \times (1.024 - 0))) + 0.215 = 0.417$<br>$DW_{diff}$: 0.589 − 0.215 = 0.374<br>$coef_{diff}$: 2.232<br>$coef_{modCMMVI}$: 1.024<br>$coef_{quad}$: 0<br>$DW_{deaf}$: 0.215

Table S7. Regression coefficients, percentage outgoing from two anchor and resulting disability weights using a simple linear model (according to scenario analysis 1).

Row	Health States (Ordered by Regression Coefficient; Column A)	Column A	Column B	Column C
		Coeff.	%	DW
1	Mild Vision Disorder	2.803	−25.588	0.119
2	Deafness	2.232	0.000	0.215
3	Breast Cancer (Clinically disease-free stage without permanent sequelae)	2.038	8.693	0.248
4	Severe Asthma	1.861	16.607	0.277
5	Problems of Alcohol Drinking	1.861	16.632	0.277
6	Chronic Low Back Pain	1.782	20.145	0.290
7	HIV/AIDS (seropositive, asymptomatic)	1.750	21.601	0.296
8	Mild Dementia	1.670	25.188	0.309
9	Diabetes Mellitus (uncomplicated, poorly controlled)	1.511	32.312	0.336
10	Manifest Alcoholism	1.249	44.053	0.380
11	Coronary Heart Disease, Severe Stable Angina	1.105	50.498	0.404
12	Chronic Metallic Mercury Vapor Intoxication (moderate case)	1.024	54.112	0.417
13	Colorectal Cancer (Stage of diagnosis and primary therapy)	1.023	54.183	0.418
14	Stroke, moderate impairments	0.782	64.982	0.458
15	Severe Depression	0.317	85.818	0.536
16	Delirium caused by excessive alcohol intake	0.239	89.313	0.549
17	Quadriplegia	0.000	100.000	0.589
18	Chronic Metallic Mercury Vapor Intoxication (severe case)	−0.448	120.074	0.664

Table S8. Results of integrating quadriplegia, moderate chronic metallic mercury vapor intoxication (CMMVI), and severe CMMVI into a visual analogue scale (VAS; 0: worst imaginable health state; 100: best imaginable health state) with deafness (value 78) predefined and applying exclusion criteria for outlier control.

Health State	*n*	Min DW	Max DW	DW, Mean I	II	III	IV	DW, Median I	II	III	IV	DW, Freq. V	VI
Deafness	/					0.22 (predefined)							
Moderate CMMVI	24	0.00	0.80	0.42	0.42	0.44	0.42	0.40	0.40	**0.40**	0.40	0.35	sev.
Severe CMMVI	16	0.15	0.93	0.72	0.77	0.71	0.77	0.80	0.80	**0.80**	0.80	0.85	0.80
Quadriplegia	74	0.10	1.00	0.74	0.74	0.80	0.79	0.80	0.80	**0.82**	0.82	0.80	0.90

Abbreviations: disability weight (DW), exclusion criteria (I–VI), maximum (Max), minimum (Min). I: including all mentioned values; II: excluding values mentioned by not more than 1 person; **ÍII: excluding values which are obviously wrong** (values higher than the predefined value for deafness: 0.78 (≙DW: 0.22)); IV: excluding values mentioned by not more than 1 person and which are obviously wrong; V: value with the highest frequency; VI: value with the second highest frequency; sev.: several second highest peaks: 0.60, 0.50, 0.40, 0.30; **printed in bold:** most suitable averaged values.

References

1. Salomon, J.A.; Haagsma, J.A.; Davis, A.; de Noordhout, C.M.; Polinder, S.; Havelaar, A.H.; Cassini, A.; Devleesschauwer, B.; Kretzschmar, M.; Speybroeck, N.; et al. Disability weights for the global burden of disease 2013 study. *Lancet Glob. Health* **2015**, *3*, e712–e723.
2. Schwarzinger, M.; Stouthard, M.E.; Burstrom, K.; Nord, E. Cross-national agreement on disability weights: The european disability weights project. *Popul. Health Metr.* **2003**, *1*, 9.
3. Steckling, N.; Plass, D.; Bose-O'Reilly, S.; Kobal, A.B.; Krämer, A.; Hornberg, C. Disease profile and health-related quality of life (HRQoL) using the euroqol (EQ-5D + C) questionnaire for chronic metallic mercury vapor intoxication. *Health Q. Life Outcomes* **2015**, *13*, 196.
4. Stouthard, M.E.; Essink-Bot, M.L.; Bonsel, G.J.; Barendregt, J.J.; Kramers, P.G.; Water, H.D.; Gunning-Schepers, L.J.; Maas, P.D. *Disability Weights for Diseases in The Netherlands*; Department of Public Health, Erasmus University: Rotterdam, The Netherlands, 1997.
5. Stouthard, M.E.A.; Essink-Bot, M.L.; Bonsel, G.J. Disability weights for diseases. Modified protocol and results for a western european region. *Eur. J. Public Health* **2000**, *10*, 24–34.
